# The potential of N2-modified cap analogues for precise genetic manipulation through mRNA engineering

**DOI:** 10.3389/fmolb.2023.1269028

**Published:** 2024-02-06

**Authors:** Karol Kurpiejewski, Anna Stankiewicz-Drogon, Karolina Piecyk, Eliza Rajkowska, Paulina Skrzypczyk, Jingping Geng, Edward Darzynkiewicz, Renata Grzela, Marzena Jankowska-Anyszka

**Affiliations:** ^1^ Faculty of Chemistry, University of Warsaw, Warsaw, Poland; ^2^ Division of Biophysics, Institute of Experimental Physics, Faculty of Physics, University of Warsaw, Warsaw, Poland; ^3^ Centre of New Technologies, University of Warsaw, Warsaw, Poland

**Keywords:** cap analogues, mRNA engineering, mRNA-based drugs, mRNA therapeutics, mRNA technology

## Abstract

The technology of mRNA-based drugs is currently being intensively developed and implemented. Medical products of this type are already being used as viral vaccines and could potentially find application in a wide range of diseases. The tremendous interest in mRNA is due to the relatively easy production process, which can be quickly adapted to meet societal needs. The properties of this molecule depend on the structure of its individual components, such as the structure of the cap at the 5ʹ end. Modifications of the cap significantly affect the translational potential and lifespan of the whole mRNA. In the current work, we present the synthesis of derivatives of cap analogues modified at the N2 position of 7-methylguanosine. In addition to the substituent at the N2 position, the derivatives had either an extended triphosphate chain, a thiophosphate modification, an added cap1-modified nucleotide or an extended linker between the substituent and 7-methylguanosine. The compounds were tested for use as translation inhibitors and as components for mRNA preparation and appeared of interest for both applications.

## 1 Introduction

Before 2020, few would have considered mRNA vaccines could become so quickly a “game-changer” in the fight against serious health and life-threatening diseases. The turning point in the perception of mRNA-based technology in medical application came with the SARS-CoV-2 virus pandemic. One of the greatest advantages of mRNA is that it can be easily modified. The improvement of stability and translational efficiency of mRNA is often achieved by modifications of the cap structure at the 5ʹ end.

The cap structure is a natural modification that consists of a 7-methylguanosine linked by a 5′–5′ triphosphate bridge to the first transcribed nucleotide (Supplementary Figure S1A) (Furuichi, 2015). Cap plays a key role in many aspects of mRNA metabolism providing protection against nucleolytic degradation and allowing mRNA recruitment to ribosome by eIF4E (Furuichi, 2015). In cells, there are three types of caps, cap0, 1 and 2. Cap0 is the simplest possible structure, bearing no additional methylation on the first transcribed nucleotide within the ribose. In mammalian cells, mRNAs are usually terminated by m^7^GpppN_m_ or m^7^GpppN1_m_N2_m_ called cap1 and cap2, respectively, where N_m_ is the 2′-*O*-methylated nucleotide (Züst et al., 2011) and the role of this modification is to differentiate between “self” and “non-self” RNA during viral infection.

It is known that factor eIF4E, when overexpressed, contributes to increased translation of proteins associated with cell division which leads to tumorigenesis (Lazaris-Karatzas et al., 1990). Modified cap analogues, most often in the form of mono- or dinucleotides, that have a high affinity for eIF4E, can act as potential translation inhibitors. Additionally, dinucleotides can be effectively used to prepare mRNA transcripts with high translational activity (Shanmugasundaram et al., 2022). Attractive analogues, which showed several times higher affinity for eIF4E, turned out to be those with aliphatic, cyclic and aromatic substituents within the exocyclic N2-amino group of N7-methylated guanine (Cai et al., 1999; Piecyk et al., 2012; 2014). It is also known that elongation of the phosphate bridge usually leads to better inhibitory properties of cap analogue. This is explained by a stronger interaction with eIF4E due to the formation of additional water-mediated hydrogen bonds and salt bridges (Rydzik et al., 2009).

Currently, the most common method for preparation of capped mRNA is incorporation of a dinucleotide by an RNA polymerase during the *in vitro* transcription (IVT) reaction. However, the existence of two free 3′-OH groups on both guanosine moieties allows transcription elongation on both sides, resulting in a translationally active product with cap structure introduced in the correct orientation of m^7^GpppG(pN)_n_ but also translationally inactive mRNA with cap in the reverse orientation of Gpppm^7^G(pN)_n_ (Pasquinelli et al., 1995). The solution to this problem came with the discovery of ARCA-type analogues (Anti-Reverse Cap Analogues) bearing an O-methyl group at the C2′ or C3′ position of 7-methylguanosine. These were the first dinucleotide cap analogues to enable incorporation almost exclusively in the correct orientation (Supplementary Figure S1B) (Grudzien-Nogalska et al., 2007a). Subsequent modifications of ARCA within the phosphate bridge showed that β-modification in the form of a thiophosphate (β-S-ARCA) increased mRNA stability while ensuring similar or increased translation efficiency (Grudzien-Nogalska et al., 2007a). Moreover, enrichment of ARCA with a benzyl substituent at the N2 position yielded mRNA with increased translational properties and higher stability *in vivo* compared to m^7^GpppG- or ARCA-capped transcripts (Kocmik et al., 2018). In our previous studies, we presented a series of N2-modified dinucleotide cap analogues without a methyl group at the 2ʹ-*O* or 3ʹ-*O* position, that were both efficiently and mostly in correct orientation incorporated into mRNA chain and resulted in a highly efficient translation (Grzela et al., 2022). Such dinucleotides have become an alternative to ARCA-type analogues.

Obtaining mRNA transcripts by IVT using dinucleotide analogues has some limitations. First, to ensure a high transcription yield, purine must be present as the first transcribed nucleotide. Second, the 2ʹ-*O* modification of the first transcribed nucleotide (cap1) prevents the incorporation of such an analogue during IVT. The novel idea of using trinucleotide analogues of cap containing such modification (m^7^GpppN_m_pN) avoids these limitations and has been already used to obtain Pfizer-BioNTech’s mRNA vaccine against SARS-CoV-2 (Ishikawa et al., 2009).

In this work, we continued our research on cap analogues modified within the exocyclic amino group. We synthesized a series of N2-modified dinucleotide analogues: triphosphate, β-S triphosphate and tetraphosphate compounds as well as three trinucleotides, bearing aromatic N2 substitution (on the m^7^G side), and evaluated their properties in biological experiments.

## 2 Methods

### 2.1 Synthesis

All presented compounds were obtained according to synthetic procedures described in detail in Supplementary Material.

### 2.2 Synthesis of luciferase encoding mRNAs

A PCR product encompassing the firefly luciferase coding sequence under the T7 RNA polymerase promoter was used as the template for the IVT reaction. Transcription buffer, 25 ng/μL dsDNA template, 0.5 mM ATP/CTP/UTP, 0.1 mM GTP, 0.5 mM dinucleotide analogue of cap (cap:GTP molar ratio was 5:1), 0.5 U/µL Ribolock ribonuclease inhibitor and 1 U/µL RNA polymerase T7 (Thermo Fisher Scientific) were added to the reaction mix (Strenkowska et al., 2016). The transcription reaction was incubated for 1 h at 37°C, followed by the addition of 0.025 U/µL DNaseI (Thermo Scientific) and incubation for 20 min at 37°C. The prepared transcripts were purified using NucleoSpin^®^ RNA Clean-Up (Macherey-Nagel) according to the manufacturer’s instructions. The integrity of the transcripts was tested on a non-denaturing 1% agarose gel and the concentration was measured spectrophotometrically.

### 2.3 Translation measurement in the RRL system

The expression level of luciferase mRNA bearing the synthesized cap analogues was measured in the Flexi Rabbit Reticulocyte Lysate System, Promega, (Strenkowska et al., 2016). The reaction mixture contained 40% Flexi RRL lysate, 0.01 mM amino acid mixture, 0.9 mM magnesium acetate (1.8 mM endogenous magnesium concentration in the lysate) and 190 mM potassium acetate. Samples were preincubated for 60 min at 30°C before the addition of mRNA. The translation reaction was carried out under the same conditions for another 60 min. The activity of the synthesized luciferase was measured using a Glomax luminometer.

### 2.4 Translation inhibition

The inhibitory activities of the new cap analogues were tested using Flexi Rabbit Reticulocyte Lysate, Promega, under conditions specified for cap-dependent translation (Kowalska et al., 2009). Briefly, the reaction mixture was pre-incubated for 60 min at 30°C, followed by the addition of a mixture of ARCA-mRNA encoding the firefly luciferase and the cap analogue under study. Samples were incubated for further 60 min at 30°C. Luciferase activity was measured using a Glomax luminometer (Promega). IC_50_ values were determined by non-linear regression analysis of the experimental data using GraphPad Prism 8. IC_50_ values are mean ± SD from at least three independent replicates.

### 2.5 Synthesis of short RNAs and cap hydrolysis with hNudt16

The annealing product of two complementary primers: 5ʹ CAG​TAA​TAC​GAC​TCA​CTA​TA**G**GGA​AGC​GGG​CAT​GCG​GCC​AGC​CAT​AGC​CGA​TCA 3ʹ and 5ʹ TGA​TCG​GCT​ATG​GCT​GGC​CGC​ATG​CCC​GCT​TCC**C**TAT​AGT​GAG​TCG​TAT​TAC​TG 3ʹ was used as a template for short RNA synthesis. Thus obtained dsDNA contained the sequence of T7 promoter and G at position +1 (bold). Capping was performed co-transcriptionally during IVT. Transcripts were purified on Oligo Clean-Up and Concentration columns (Norgen Biotek) followed by DNAzyme trimming and subsequent purification (Grzela et al., 2022). 100 ng of analogue-capped RNA was digested with 2.5 µM hNudt16 and loaded onto 15% polyacrylamide/7 M urea TBE gel as described previously (Grzela et al., 2022). To assess both capping efficiency and the amount of decapped product, a densitometric analysis with the use of Image Lab software (Bio-Rad) was applied. Decapping was calculated as the per cent loss in the capped band after addition of the enzyme. The data represent the means ± SD from three experiments.

### 2.6 Statistical analysis

All statistical analyses were performed with GraphPad Prism 8 software. One-way ANOVA with *post hoc* Turkey test was used. Statistical significance in *p*-value is denoted in asterixes ****p < 0.0001, ***p < 0.001, and **p < 0.01 and the data are shown as mean ± SD.

## 3 Results

### 3.1 Chemistry

**FIGURE 1:**
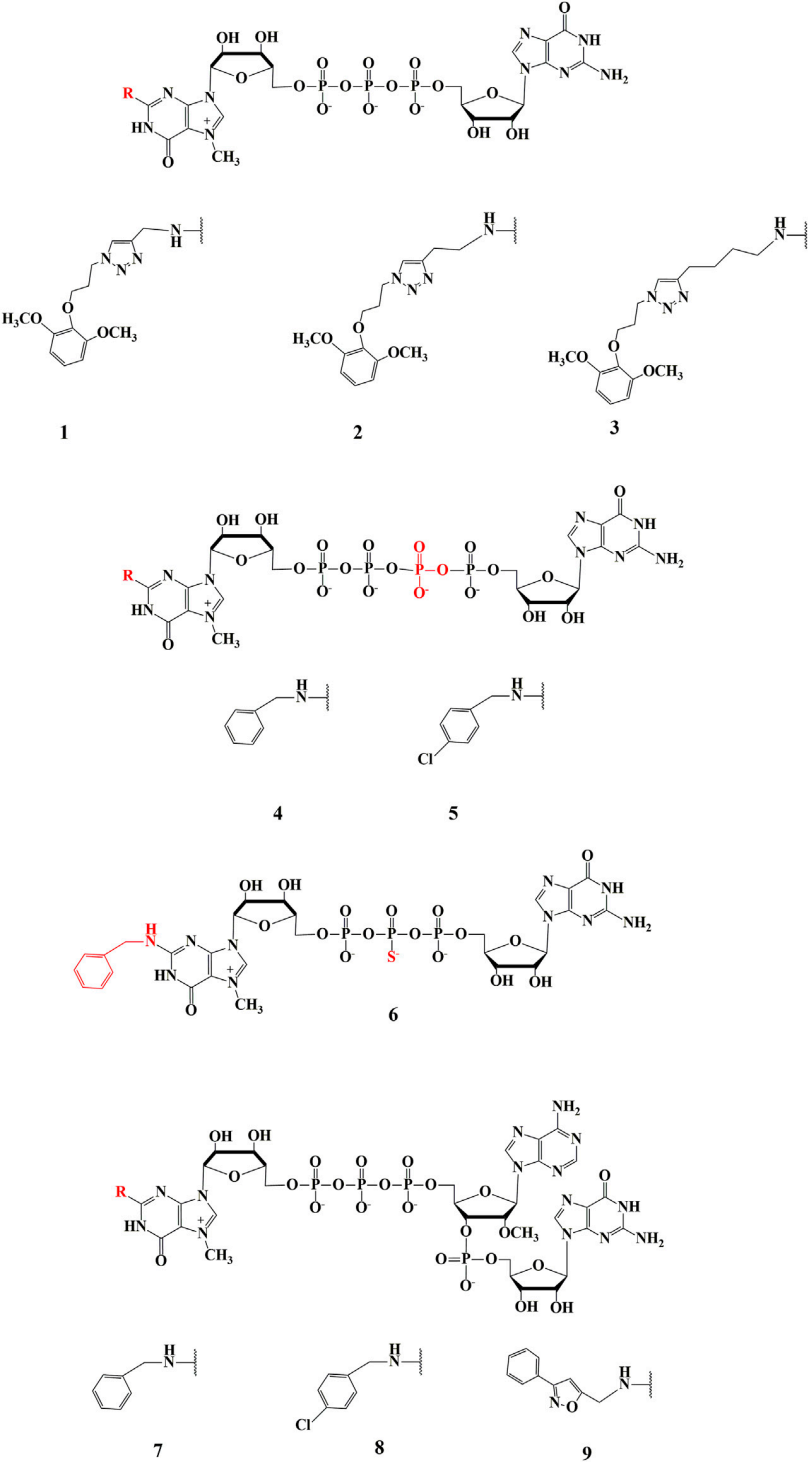
Structure of new cap analogues, modified at N2 position of m7-guanosine. **1**—(4-(diOCH_3_-bn)-tz-CH_2_)^2^m^7^GpppG, **2**—(4-(diOCH_3_–bn)-tz-(CH_2_)_2_)^2^m^7^GpppG, **3**—(4-(diOCH_3_–bn)-tz-(CH_2_)_4_)^2^m^7^GpppG, **4**—(bn^2^m^7^GppppG, **5**—(4-Cl-bn)^2^m^7^GppppG, **6**—bn^2^m^7^Gpp_s_pG, **7**—bn^2^m^7^GpppA_m_pG, **8**—(4-Cl-bn)^2^m^7^GpppA_m_pG, **9**—(4-bn-isx)^2^m^7^GpppA_m_pG.

Our current research concerns the synthesis and biological studies of three groups of N2-modified cap analogues. The first group consists of dinucleotides modified only within the exocyclic amino group of the m^7^G moiety (Figure 1A) with a modified triazole ring attached to N2 position via aliphatic linkers of different lengths. The second group consists of dinucleotide analogues modified both at the N2 position of 7-methylguanosine with aromatic substituents and within the 5ʹ-5ʹ bridge in the form of tetraphosphate or β-thiophosphate (Figures 1B, C). The last group consists of trinucleotides containing modified m^7^G moiety in the N2 position with aromatic substituents (benzyl, p-chlorobenzyl and isoxazole) and 2ʹ-O-methylated adenosine as the first transcribed nucleotide (characteristic for cap1) connected via 3ʹ,5ʹ-phosphodiester bond to guanosine (R^2^m^7^GpppA_m_pG shown in Figure 1D).

The synthesis of N2-modified dinucleotide cap analogues has already been described (Piecyk et al., 2015; Grzela et al., 2022). Therefore, compounds 1-3 were obtained in an analogous procedure, with compound 1 synthesized again as a reference for biological studies.

Tetraphosphate analogues (4–5) were obtained using a method for the synthesis of a simpler analogue such as m^7^GppppN (Rydzik et al., 2009). This method requires the preparation of two 5ʹ-diphosphate subunits (GDP and m^7^GDP), one of which must be converted to an imidazole derivative using Mukaiyama method (Mukaiyama and Hashimoto, 1972) to increase the susceptibility to nucleophilic attack of the second subunit in the coupling reaction with a ZnCl_2_ catalyst under anhydrous conditions (Kadokura et al., 1997). In the course of our research, N2-substituted 7-methylguanosine 5ʹ diphosphate (7) (R^2^m^7^GDP) and an imidazole derivative of guanosine 5ʹ diphosphate (im-GDP), were synthesized and subjected to a coupling reaction to obtain a series of tetraphosphate N2-modified dinucleotides.

Compound (6) was obtained analogous to the procedure reported for the synthesis of m^7^Gpp_s_pG (Kowalska et al., 2008). In the first step, 5ʹ monophosphate of N2-benzyl, 7-methylguanosine (bn^2^m^7^GMP), was obtained and converted to its imidazole derivative (im-bn^2^m^7^GMP). Thus prepared compound was subjected to a coupling reaction using triethylammonium thiophosphate in the presence of ZnCl_2_ catalyst to obtain the diphosphate derivative bn^2^m^7^Gpp_S,_ which was further subjected to the coupling reaction with an imidazole derivative of GMP yielding the product in the form of two diastereoisomers. Since attempts to separate each isomer were unsuccessful, a mixture of diastereoisomers was used for biological experiments.

Trinucleotide analogues of cap (7–9) were obtained in a multistep synthesis, where in the final reaction, the two nucleotide subunits of im-R^2^m^7^GDP and pA_m_pG were linked together in a coupling reaction analogous to the one used for preparation of dinucleotides. The im-R^2^m^7^GDP subunit was prepared according to previously described methods. The pA_m_pG dinucleotide was synthesized in three steps using the amidophosphite method (Senthilvelan et al., 2021). In the first step, coupling reactions of the commercially available 5ʹ-O-dimetoxytrityl-N-benzoyl-2ʹ-O-methyladenosine 3ʹ-cyanoethyl phosphoramidite with N2-iso butyryl-2ʹ,3ʹ-isopropylideneguanosine were carried out according to the protocol (Eisenführ et al., 2003). The coupling reactions were conducted in acetonitrile and the presence of 5-(benzylthio)-1H-tetrazolium as activator while the oxidation was carried out using iodine to obtain the appropriately protected A_m_pG dinucleotide. In a subsequent step, the protecting group was removed from the 5ʹ-OH position using dichloroacetic acid while the ketal protection was removed using trifluoroacetic acid. Thus the indirectly deprotected dinucleotide was phosphorylated at 5ʹ-OH position by the Yoshikawa method (Yoshikawa et al., 1969), and finally deprotection was performed using an aqueous ammonia solution to obtain the desired dinucleotide pA_m_pG.

All dinucleotides and trinucleotides were isolated from the reaction mixtures and purified by ion-exchange chromatography described in Methods (Supplementary Material). The structure and homogeneity of each compound was confirmed by HPLC, high-resolution mass spectrometry with positive electrospray ionisation (HRMS-ESI) and ^1^H and ^31^P NMR.

### 3.2 Inhibition

**FIGURE 2:**
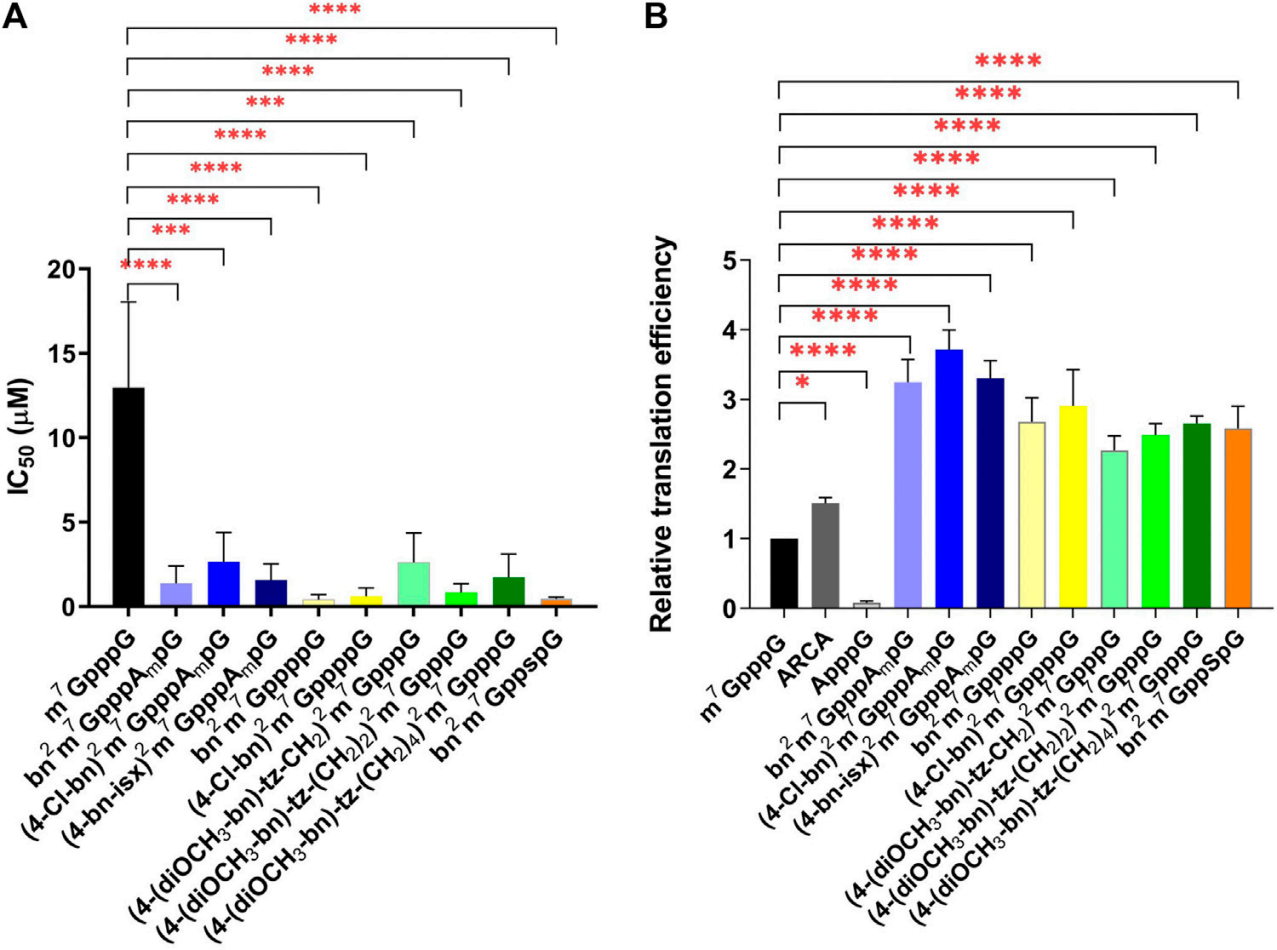
Properties of new N2-modified cap analogues in RRL. **(A)** Cap analogues as translational inhibitors. The graph represents inhibition of translation of ARCA-capped mRNA encoding firefly luciferase in RRL upon addition of cap analogues. IC_50_ stands for the cap analogue concentration that inhibits luciferase activity by 50%. IC_50_ values were determined by non-linear regression analysis of the experimental data using GraphPad Prism 8 and are presented as mean ± SD of at least three independent replicates. **(B)** Translational properties of mRNAs capped with N2-modified analogues. Luciferase activity presented in the graph reflects the level of translation in RRL of analogue-capped mRNA encoding firefly luciferase. Data are presented as mean ± SD of at least three independent experiments.

The protein biosynthesis process depends on the delivery of the information encoded on the mRNA to ribosomes by the eIF4E protein. The efficiency of this process is related to the affinity of eIF4E to the cap structure located at the 5ʹ end of the mRNA. The addition of a free cap analogue with high affinity for eIF4E impairs protein synthesis. Therefore, new compounds are routinely tested for their ability to inhibit translation. For this purpose, we used an extracellular translation system from rabbit reticulocytes (RRL). We added the ARCA-bearing mRNA encoding firefly luciferase to an RRL extract containing all factors necessary for protein synthesis and measured the bioluminescence level. To the subsequent sample, we added increasing amounts of newly synthesized cap analogues and observed changes in bioluminescence relative to the control sample without free cap analogue. Compounds bn^2^m^7^GppppG (4) and (4-Cl-bn)^2^m^7^GppppG (5) turned out to be the most effective inhibitors, with IC_50_ of 0.43 ± 0.16 and 0.60 ± 0.29, respectively (Table 1; Figure 2A). These compounds are tetraphosphates of the derivatives described previously (Grzela et al., 2022), that have shown strong inhibition of protein biosynthesis. Here, an even stronger inhibitory effect was observed. This can be explained by the presence of an additional phosphate in the triphosphate chain that enhance the interaction between cap and eIF4E.

**TABLE 1:** Biological proprieties of novel cap analogues and analogue-capped mRNA.

Cap analogue	IC50 ± SD (µM)	Relative translation efficiency ±SD	Relative cap-dependent translation efficiency	Capping ±SD (%)	Decapping ±SD (%)	References
m^7^GpppG	8.94 ± 0.86	1	1	74.30	45.74 ± 3.42	Grzela et al. (2018)
bn^2^m^7^GpppG	0.61 ± 0.04	3.30 ± 0.77	3.52 ± 0.82	80.50	29.05 ± 3.72	Grzela et al. (2022)
(4-Cl-bn)^2^m^7^GpppG	0.98 ± 0.13	3.29 ± 0.74	3.52 ± 0.73	88.20	26.45 ± 7.34	Grzela et al. (2018)
(4-bn-isx)^2^m^7^GpppG	0.57 ± 0.06	3.37 ± 0.84	3.51 ± 0.82	88.50	33.25 ± 7.16	Grzela et al. (2022)
Newly Synthesized Derivatives						
m^7^GpppG	12.98 ± 5.07	1	1	70.60 ± 4.91	52.77 ± 10.82	this work
ARCA	nd	1.51 ± 0.08	1.56 ± 0.10	61.87 ± 3.60	6.70 ± 1.85	this work
ApppG	nd	0.08 ± 0.03	0	94.57 ± 1.78 (GpppG)	87.87 ± 4.69 (GpppG)	this work
bn^2^m^7^GpppA_m_pG (7)	1.38 ± 1.02	3.25 ± 0.32	3.43 ± 0.40	90.22 ± 7.65	7.62 ± 2.48	this work
(4-Cl-bn)^2^m^7^GpppA_m_pG (8)	2.66 ± 1.73	3.72 ± 0.28	3.94 ± 0,36	91.51 ± 3.81	6.73 ± 4.20	this work
(4-bn-isx)^2^m^7^GpppA_m_pG (9)	1.57 ± 0.95	3.31 ± 0.25	3.49 ± 0.32	nd	nd	this work
bn^2^m^7^GppppG (4)	0.43 ± 0.28	2.68 ± 0.34	2.82 ± 0.41	88.55 ± 4.60	37.40 ± 14.99	this work
(4-Cl-bn)^2^m^7^GppppG (5)	0.60 ± 0.50	2.91 ± 0.52	3.07 ± 0.58	92.00 ± 7.21	55.07 ± 6.73	this work
(4-(diOCH_3_-bn)-tz-CH_2_)^2^m^7^GpppG (1)	2.61 ± 1.74	2.27 ± 0.20	2.36 ± 0.25	85.63 ± 2.10	11.50 ± 1.50	this work
(4-(diOCH_3_-bn)-tz-(CH_2_)_2_)^2^m^7^GpppG (2)	0.85 ± 0.51	2.49 ± 0.16	2.60 ± 0.17	85.77 ± 7.00	29.70 ± 5.40	this work
(4-(diOCH_3_-bn)-tz-(CH_2_)_4_)^2^m^7^GpppG (3)	1.73 ± 1.37	2.66 ± 0.09	2.83 ± 0.10	82.13 ± 2.72	12.40 ± 2.42	this work
bn^2^m^7^GppspG (6)	0.45 ± 0.11	2.58 ± 0.28	2.75 ± 0.31	78.00 ± 4.70	2.60 ± 1.39	this work

Compound bn^2^m^7^Gpp_s_pG (6) appeared as potent translational inhibitor as bn^2^m^7^GppppG (4) with an IC_50_ of 0.45 ± 0.05 (Table 1; Figure 2A). For the trinucleotide analogues (bn^2^m^7^GpppA_m_pG (7), (4-Cl-bn)^2^m^7^GpppA_m_pG (8), (4-bn-isx)^2^m^7^GpppA_m_pG (9)), the IC_50_ values ranged from 1.38 ± 0.59 to 2.66 ± 1.0, while for compounds with longer linkers [(4-(diOCH_3_-bn)-tz-CH_2_)^2^m^7^GpppG (1), (4-(diOCH_3_-bn)-tz-(CH_2_)_2_)^2^m^7^GpppG (2), (4-(diOCH_3_-bn)-tz-(CH_2_)_4_)^2^m^7^GpppG (3)] from 0.85 ± 0.30 to 2.61 ± 1.0 (Table 1; Figure 2A). Although these scores are higher, still the studied compounds represent more potent inhibitors of translation than m^7^GppppG.

### 3.3 Translational properties of mRNA capped with newly synthesized cap analogues

An extracellular translation system from RRL was used to assess the translational properties of mRNAs containing newly synthesized cap analogues. Analogue-capped mRNAs encoding luciferase were added to the extracts and protein activity was measured. The highest levels of luciferase synthesis were provided by mRNAs with trinucleotide derivatives bn^2^m^7^GpppA_m_pG (7), (4-Cl-bn)^2^m^7^GpppA_m_pG (8), and (4-bn-isx)^2^m^7^GpppA_m_pG (9). The translation level of mRNA capped with these derivatives was 3.31, 3.25 and 3.72 times higher than that of m^7^GpppG-mRNA, respectively (Table 1; Figure 2B).

Transcripts bearing derivatives with a tetraphosphate bridge, bn^2^m^7^GppppG (4) and (4-Cl-bn)^2^m^7^GppppG (5), showed lower translational properties compared to their initial compounds bn^2^m^7^GpppG and (4-Cl-bn)^2^m^7^GpppG (Table 1). RNA capped with compounds that differ in linker length (4-(diOCH_3_-bn)-tz-CH_2_)^2^m^7^GpppG (1), (4-(diOCH_3_-bn)-tz-(CH_2_)_2_)^2^m^7^GpppG (2), (4-(diOCH_3_-bn)-tz-(CH_2_)_4_)^2^m^7^GpppG (3) show very similar level of translation, e.g., around 2.2–2.6 times higher than observed for m^7^GpppG-RNA (Table 1). RNA bearing cap analogue with a thiophosphate modification bn^2^m^7^GppspG (6) showed 2.6 times higher translation than m^7^GpppG-RNA (Table 1; Figure 2B).

### 3.4 Capping efficiency of RNA

**FIGURE 3:**
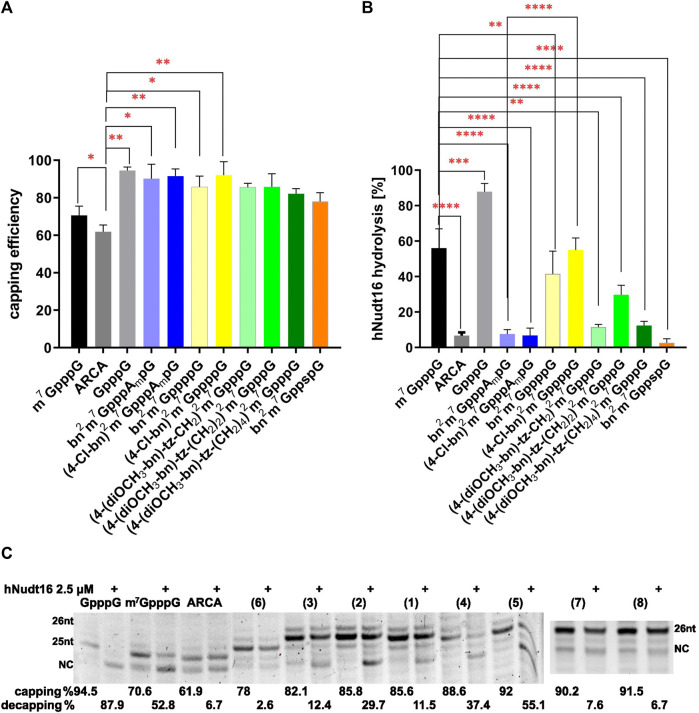
Efficiency and orientation of incorporation of N2-modified cap analogues into RNA. **(A)** The level of RNA 5ʹ capping with new cap analogues. The graph represents the percentage of capped RNA obtained during IVT reaction, calculated from densitometric analysis. Capping was calculated as percent of capped product in samples not subjected to hydrolysis. **(B)** The level of cap hydrolysis with hNudt16. The graph represents the per cent loss in the capped fraction of the sample after 30 min hydrolysis with hNudt16. Data represent mean ± SD of three independent experiments and were analyzed using GraphPad Prism 8 (GraphPad Software, San Diego California, United States) statistical analysis software. Statistical analysis were performed using ordinary one-way ANOVA with Turkey test. Statistical significance between the RNA with modified caps and m^7^GpppG-capped RNA is denoted by a value of ****p < 0.0001, ***p < 0.001, and **p < 0.01. Otherwise, the differences are not statistically significant. **(C)** Exemplary gel electrophoretic analysis of 5ʹ cap hydrolysis with hNudt16. NC denotes product without the cap (not capped and/or decapped). Capping and decapping levels were estimated by densitometric analysis of bands using ImageLab software (Bio-Rad). The data represent mean ± SD of three independent experiments.

To assess the incorporation of cap analogues, we used short RNAs that, after synthesis, were visualized on a UREA-PAGE gel. Such gels have a high capacity to separate products that differ in length by one nucleotide. The mRNA chain without the cap analogue has a length of 24 nt and is denoted by NC in Figure 3. When the cap analogue is attached, the short RNA migrates higher depending on whether a dinucleotide (25 nt) or a trinucleotide (26 nt) is attached. Residual products are also visible on the gel, which is due to the properties of the RNA polymerase, which has the ability to fall off the template before the product is completed or to further attach nucleotides off-template. Control caps: GpppG, m^7^GpppG, and ARCA showed expected incorporation efficiencies (Grzela et al., 2022) of 94.57%, 70.60% and 61.87%, respectively (Figure 3; Table 1). The trinucleotides (bn^2^m^7^GpppA_m_pG (7) and (4-Cl-bn)^2^m^7^GpppA_m_pG (8)) had a very high incorporation efficiency of 90.22% and 91.51%, similarly bn^2^m^7^GppppG (4) and (4-Cl-bn)^2^m^7^GppppG (5) (88.55% and 92.00%, respectively) while the percentage of incorporation of remaining dinucleotide caps into mRNA ranged from 78.00% to 85.77% (Figure 3; Table 1).

### 3.5 Orientation of incorporation

Next, we estimated the orientation of cap incorporation into RNA. For this purpose, we used an assay we developed with the enzyme hNudt16 (Grzela et al., 2022). In a certain concentration range, this enzyme performs the hydrolytic reaction only when the RNA at the 5ʹ end exposes unmethylated guanosine (Grzela et al., 2018; Chrabąszczewska et al., 2021). Given that the cap is asymmetric concerning methylation at position 7 of guanosine, the rate of hydrolysis reflects its orientation of incorporation.

Among the tested analogues, only 7.62% of the bn^2^m^7^GpppA_m_pG-capped RNA and 6.73% of the (4-Cl-bn)^2^m^7^GpppA_m_pG-capped RNA was hydrolyzed by the hNudt16 (Figure 3; Table 1). Such a result indicates the incorporation of the analogue only in the correct orientation. A similar level of hydrolysis was observed for bn^2^m^7^Gpp_s_pG-capped RNA (Figure 3; Table 1). However, the orientation of this compound incorporation cannot be assessed, since the presence of a thiophosphate modification in the triphosphate bridge prevents the hydrolysis reaction from proceeding. RNAs capped with compounds (4-(diOCH_3_-bn)-tz-CH_2_)^2^m^7^GpppG (1) and (4-(diOCH_3_–bn)-tz-(CH_2_)_4_)^2^m^7^GpppG (3) undergo hNudt16 hydrolysis to a small extent (11.5%–12.4%), leading to the conclusion that these analogues are built into RNA mostly in the correct orientation, while capping with (4-(diOCH_3_–bn)-tz-(CH_2_)_2_)^2^m^7^GpppG (2) is incorrect in about 30% (Figure 3; Table 1).

Given that the hNudt16 hydrolysis assay was developed for triphosphate-chain dinucleotides, we checked its fidelity for control tetraphosphate-chain dinucleotides. We used m^7^GppppG and m^7^Gppppm^7^G for the reaction. Hydrolysis of the first analogue showed the presence of GMP and m^7^GTP products. The second of studied analogues, m^7^Gppppm^7^G, did not undergo hydrolysis at the hNudt16 concentration tested. This confirms the validity of the statement that the enzyme preferentially attacks unmethylated guanosine. Therefore, we can conclude that the hydrolysis of dinucleotides with a tetraphosphate bridge proceeds in the same way as for triphosphate compounds.

The hydrolysis of RNA capped with analogues bearing tetraphosphate chain (bn^2^m^7^GppppG (4) - and (4-Cl-bn)^2^m^7^GppppG (5)) proceeded at a similar rate as that for m^7^GpppG-RNA, indicating two possible orientations of incorporation of these analogues into RNA chain (Figure 3; Table 1).

## 4 Discussion

Despite the many drugs available on the pharmaceutical market, we are still unable to cure many serious diseases. Efficacy, safety and short time to market are important criteria for drug development. Response time to an emerging threat is particularly important in the case of viral diseases, that can take on a pandemic spread. Given global temperatures rise, melting glaciers releasing old pathogens or the increasing transmission of pathogens from animals to humans, the danger of new diseases emerging is high (Alempic et al., 2023). Therapeutic mRNA molecule has brought new hope, as its production process is quick and easily adjustable for many different therapeutic applications (Li et al., 2023; Yu et al., 2023). By changing the sequence, any protein of therapeutic interest can be obtained. As a result, the waiting time for a new medicine, or a medicine tailored to the patient, is significantly reduced.

For the efficient production of a protein encoded on mRNA, the individual components of the carrier molecule such as the cap structure, 5ʹ and 3ʹ UTRs, modified nucleotides or poliA are important. The cap structure is a 7-methyloguanosine attached via a triphosphate bond to the RNA chain. Intensive research work has led to the introduction into the biotechnology market of several synthetic analogues of particular interest: m^7^GpppG, ARCA, β-S-ARCA, and CleanCap (Grudzien-Nogalska et al., 2007a; Grudzien-Nogalska et al., 2007b; Henderson et al., 2021).

However, this does not exhaust the possible modifications that can give the cap structure a new quality. New analogues modified at the N2 position of 7-methylguanosine have recently been synthesized (Grzela et al., 2022). The positioning of benzene substituents at the N2 7-methylguanosine allowed efficient synthesis of protein from modified mRNA at levels more than three times higher than in the case of RNA bearing the standard m^7^GpppG analogue. The new caps also had an extremely interesting feature, since some substituents at the N2 position of 7-methylguanosine prevented the polymerase from attacking the methylated guanosine. This resulted in the elongation of the RNA chain from the unmethylated guanosine only, so that the entire transcript had cap incorporated in the correct orientation.

The aim of this study was the small-scale synthesis of new cap analogues that would simultaneously carry N2 modifications of 7-methylguanosine and the following other modifications: a) added adenine with 2ʹ-*O* ribose methylation at position +1, b) triphosphate chain extended by one phosphate, c) added thiophosphate modification in the triphosphate chain, d) altered linker between 7-methylguanosine and the substituent. The addition of these modifications is known to provide the following features: recognition of the mRNA molecule as a “self” (Vladimer et al., 2014; Miedziak et al., 2020), enhanced affinity for eIF4E (Strenkowska et al., 2010), protection against the activity of decapping enzymes and in consequence extended lifespan of mRNA in the cell (Grudzien-Nogalska et al., 2007a), and different structural arrangement of the derivative relative to binding pocket of eIF4E. Since synthesizing compounds with the described modifications is difficult and challenging, we aimed at a small-scale synthesis, that would allow for initial characterization of the analogues and selection of most promising ones for further studies.

Caps are being investigated as translation inhibitors and as a component of mRNA molecule. We characterized the newly obtained compounds in terms of their inhibitory properties using an *in vitro* translation system. Among the analogues tested, there were three that showed very strong inhibitory properties. The results obtained are as expected, since these compounds are derivatives of the analogues described in (Grzela et al., 2022), which have already shown strong inhibitory properties on protein biosynthesis. In the case of aforementioned new derivatives, the inhibitory potential was even stronger than in the case of the initial compounds. Two of these compounds: bn^2^m^7^GppppG (4) and (4-Cl-bn)^2^m^7^GppppG (5) had, in addition to the modification at N2 7-methylguanosine, the triphosphate chain extended to four phosphates, that is known to enhance the interaction between cap and eIF4E. The third analogue was bn^2^m^7^Gpp_s_pG, a compound with modification at N2 7-methylguanosine and with a thiophosphate group in the triphosphate chain. This result is very important, as an analogue of this type is characterized by its resistance to decapping enzymes and can therefore be stable for long periods of time. However, it should be noted, that we studied the compound 6 as a mixture of stereoisomers due to the difficulty in separating them. All these compounds deserve special attention in the development of anticancer drugs based on inhibitors of the translation process.

We then focused on the characterization of caps as components of the mRNA molecule. We assessed their incorporation efficiency into mRNA. Trinucleotide analogues had the highest incorporation efficiency, exeeding 90%. All other compounds modified at the N2 position of 7-methylguanosine had a fairly high percentage of incorporation into mRNA. As in the previous study (Grzela et al., 2022), compound (4-(diOCH_3_–bn)-tz-CH_2_)^2^m^7^GpppG (1) showed low levels of hydrolysis by the hNudt16 enzyme, indicating its correct incorporation orientation. We observed a similar low level of decapping for a derivative of this compound, analogue (4-(diOCH_3_–bn)-tz-(CH_2_)_4_)^2^m^7^GpppG (3). The result for RNA containing trinucleotide derivatives was even better. In contrast, derivatives with an extended triphosphate chain behaved similarly to the standard compound m^7^GpppG, which incorporates into mRNA in two orientations. This indicates that these derivatives should be developed into inhibitors rather than mRNA elements. Considering both the incorporation efficiency and its orientation, trinucleotide derivatives are ideal compounds for the preparation of therapeutic mRNA. During IVT reaction with these compounds, fewer by-products are formed due to incomplete cap incorporation or the presence of an RNA fraction with a reversibly attached cap. These factors have a significant impact on the quality of the therapeutic RNA molecule and on the subsequent cellular response to the introduced transcript.

Finally, we tested the functionality of the new derivatives attached to 5ʹ end of mRNA in an *in vitro* translation assay. The highest values were achieved by mRNAs containing trinucleotide caps. However, these values were almost the same as for the initial dinucleotide compounds modified at the N2 position of 7-methylguanosine (bn^2^m^7^GpppA_m_pG (7) vs. bn^2^m^7^GpppG, (4-Cl-bn)^2^m^7^GpppA_m_pG (8) vs. (4-Cl-bn)^2^m^7^GpppG, (4-bn-isx)^2^m^7^GpppA_m_pG (9) vs. (4-bn-isx)^2^m^7^GpppG). Only one of these compounds, (4-Cl-bn)^2^m^7^GpppA_m_pG (8), presented more effective translation than its original counterpart. It is well known, the 2ʹ-*O* ribose modification present in the trinucleotide analogues is important for proper mRNA recognition by the immune system, while the extracellular assay is not apt to evaluate the cellular response (Züst et al., 2011). Unfortunately, we were unable to perform experiments in the cells due to the small-scale of compounds synthesis. Still, the set of basic experiments we performed, allowed us to select trinucleotide derivatives modified at the N2 position of 7-methylguanosine as promising components for the preparation of mRNA. In the near future, we plan to optimize the process of synthesis of these analogues to obtain quantities that would allow tests in a cellular system and further administration of modified transcripts to animals.

## Data Availability

All relevant data is included in the article. There is no data that should be deposited in any repository. Any further questions can be directed to the respective authors, as indicated in the article.
