# Supplementary material for: The potential of N2-modified cap analogues for precise genetic manipulation through mRNA engineering
Source: Front Mol Biosci. 2024 Feb 6;10:1269028. doi: 10.3389/fmolb.2023.1269028 (PMC10878308; doi:10.3389/fmolb.2023.1269028)
Supplement: Supplementary file 1 [file DataSheet1.PDF]

Supplementary material to: The potential of N2-modified cap analogues for precise genetic manipulation through mRNA engineering

Karol Kurpiejewski[1], Anna Stankiewicz-Drogon[2], Karolina Piecyk[1], Eliza Rajkowska[1], Paulina Skrzypczyk[1], Jingping Geng[3], Edward Darzynkiewicz[3], Renata Grzela[2*], Marzena Jankowska-Anyszka[1*]

[1]Faculty of Chemistry, University of Warsaw, 02-093 Warsaw, Poland
[2]Division of Biophysics, Institute of Experimental Physics, University of Warsaw, 02-093, Poland
[3]Centre of New Technologies, University of Warsaw, 02-093, Poland

*** Correspondence:**
Renata Grzela rgrzela@fuw.edu.pl
Marzena Jankowska-Anyszka marzena@chem.uw.edu.pl

Synthesis of cap analogues

General information: Chemical reagents and starting materials, including fully protected 2′-O-methyladenosine phosphoramidite were purchased from commercial sources. P-Imidazolides of 5′-mono and diphosphate guanosine (im-GMP, im-GDP), fully protected N2-isobutyryl-2′,3′-isopropylidene-guanosine were synthesized based on previously described protocols (Eisenführ et al. 2003; Grzela et al. 2022). All obtained compounds (**1-9**) were purified by ion exchange chromatography on DEAE-Sephadex (A-25, HCO^{3-} form) using a linear gradient of triethylammonium bicarbonate (TEAB), pH 7.6, in water. Fractions containing the desired product was combined, evaporated under reduced pressure with repeated additions of 96% ethanol and then lyophilizate to obtain TEA salt of the product as a fine white powder. The remaining di- and trinucleotides were isolated from the reaction mixtures by semi-preparative RP-HPLC SUPELCOSIL™ on LC-18-DB column (21.2 × 250 mm, flow rate 2,2 ml/min) with a linear gradient of methanol from 0% to 50% (v/v) in 0.05 M ammonium acetate (pH 5.9). UV detection was performed at 254 nm and isolated from the eluate by repeated freeze-drying. The structure and homogeneity of each compound was confirmed by re-chromatography by RP-HPLC SUPELCOSIL™ LC-18-T HPLC column (4.6 × 250 mm, flow rate 1.0 ml/min) with a linear gradient of 0–50% of methanol in 0.05 M ammonium acetate buffer (pH 5.9). High-resolution mass spectrometry were recorded with Micromass Q–TOF Premier using positive electro spray ionization (HRMS-ESI). NMR spectra were recorded by Agilent 600 MHz DDR2.

General synthetic procedure for dinucleotide, tetraphosphate cap analogues modified at the N2 position (1-5)

To dissolved of imidazole derivative of guanosine 5′-diphosphate (2 eq.) in anhydrous DMF, 7-methylguanosine 5′-diphosphate or 7-methylguanosine 5′-monophosphate modified at the N2 position (obtained according to (Kocmik et al. 2018)) (1 eq.) and anhydrous $ZnCl_2$ (10 eq.) were added and allowed to stir vigorously for 24 h, at RT. The reaction was quenched by adding an aqueous solution of EDTA in disodium salt (73 mg/1 mL). The resulting dinucleotide product

(**1-5**) were isolated from the mixture on DEAD-Sephadex (gradient elution 0–1.0 M TEAB) and purified by semi-preparative RP HPLC (gradient elution 0–50% MeOH in 0.05 M ammonium acetate buffer pH 5.9) to afford—after evaporation and repeated freeze-drying from water—ammonium salt of dinucleotide cap analogues. The reaction yields ranged from 18-43%.

P1-N2-{1-[3-(2,6-dimethoxyphenoxy)propyl]-1H-1,2,3-triazol-4-yl}methylene-7-methylguanosine-P3-guanosine 5′,5′-triphosphate (1)

29 mg (0.026 mmoL), 43%, ammonium salt; ^{1}H NMR (400 MHz, D_2O) δ 8.80 (s, 1H, H8), 8.09 (s, 1H, H8), 8.00 (s, 1H, triazole), 6.90 (t, 1H, *J*=8.817), 6.45 (d, 2H, *J*=8.457), 5.80 (d, 1H, *J*=2.09), 5.77 (d, 1H, *J*=6.25), 4.65–4.57 (m, 6H, H2′, H2′, CH_2-triazole-CH_2-), 4.49-4.47 (m, 2H, H3′, H3′), 4.45-4.42 (m, 2H, H4′, H4′), 4.39-4.30 (m, 4H, H5′, H5′, H5″, H5″), 3.94 (s, 3H, CH_3), 3.87-3.75 (m, 2H, CH_2-CH_2-CH_2-Ph), 3.66 (s, 6H, 2xOCH_3), 2.32-2.29 (m, 2H, triazole-CH_2-CH_2-CH_2-Ph); ^{31}P NMR (162 MHz, D_2O) δ -11.76 (2P, Pα,γ), -23.13 (1P, Pβ); D_2O HRMS (ES+) m/z: (M+H)+: 1078.22217, calculated for $C_{35}H_{47}N_{13}O_{21}P_3^{+}$: 1078.22168.

^{1}H NMR

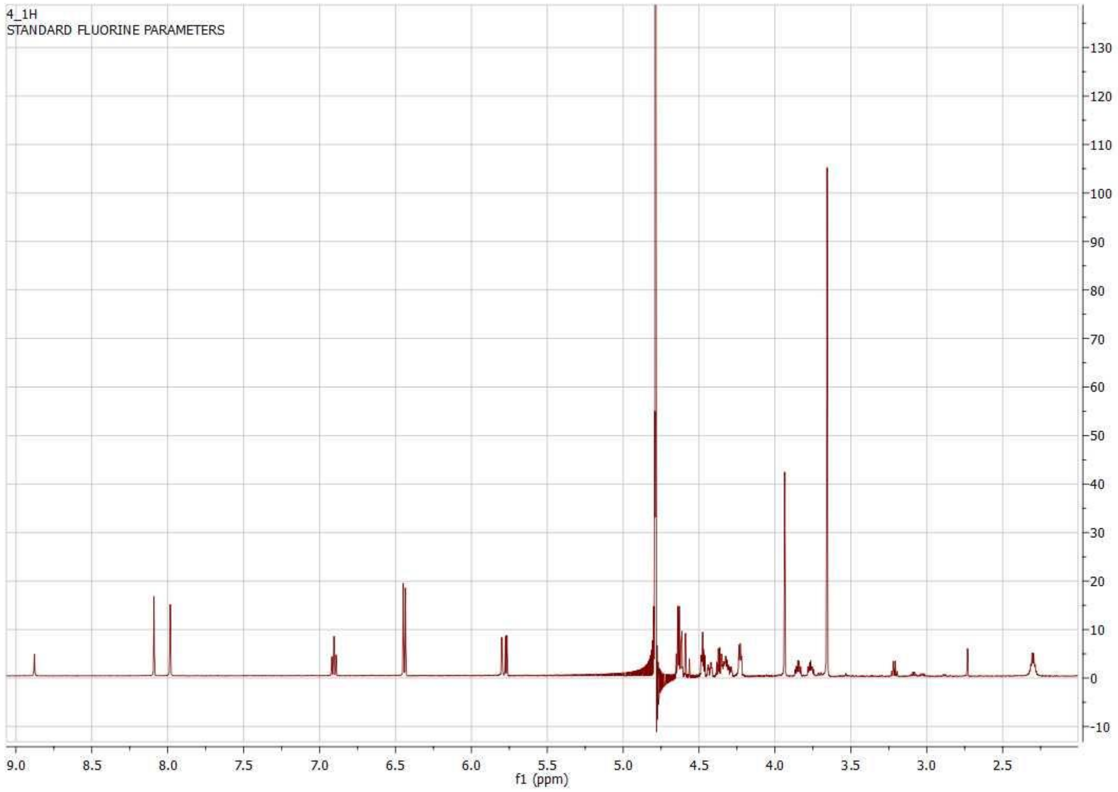

^{31}P NMR

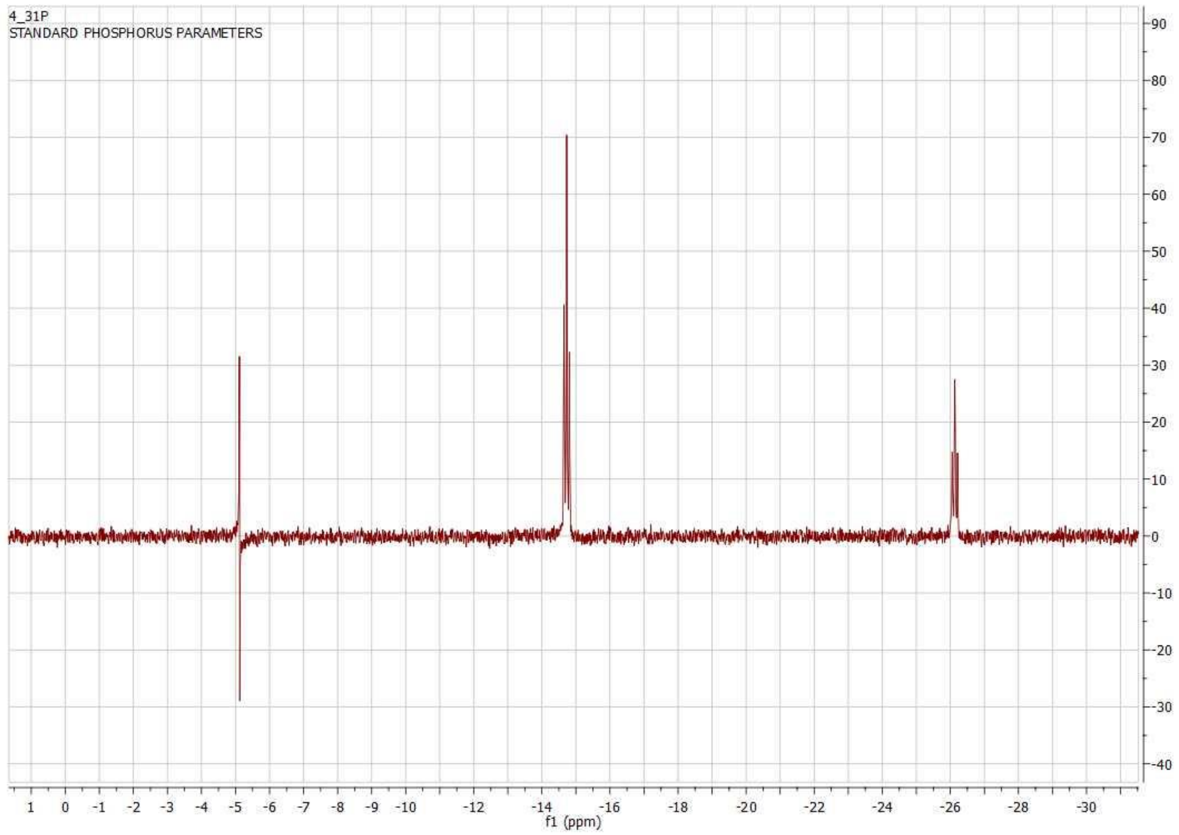

HRMS (ES+)

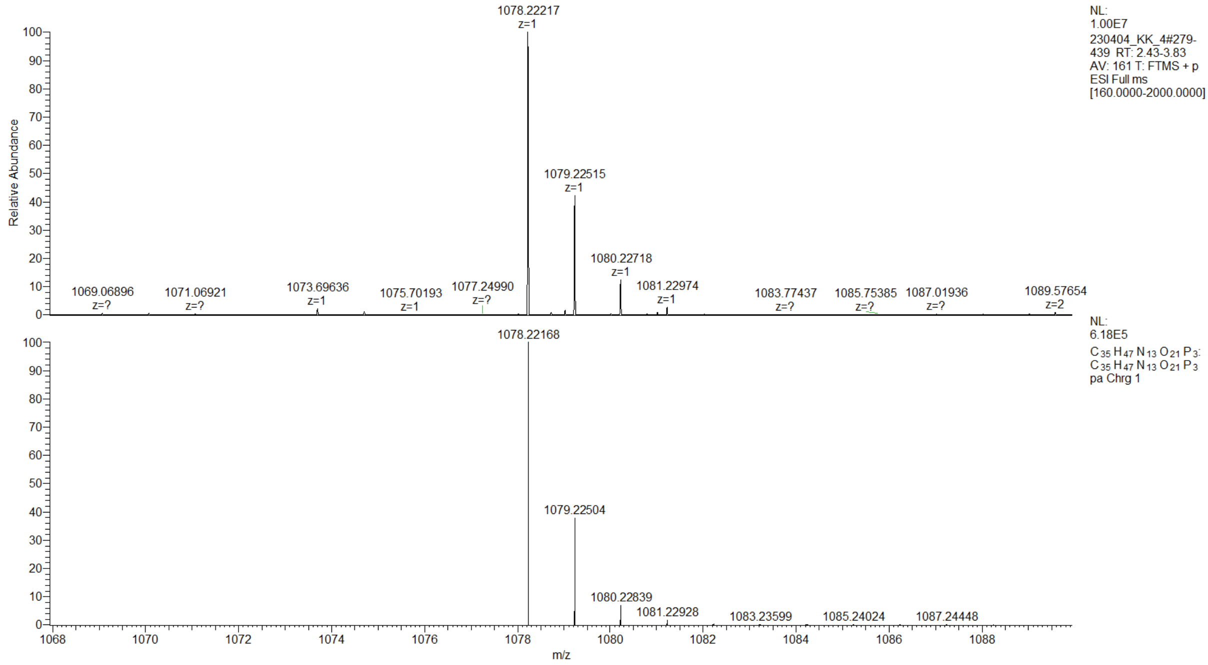

P1-N2-{1-[2-(2,6-dimethoxyphenoxy)propyl]-1H-1,2,3-triazol-4-yl}ethylene-7-methylguanosine-P3-guanosine 5′,5′-triphosphate (2)

8 mg (0.074 mmoL), 18 %, ammonium salt; ^{1}H NMR (600 MHz, D_2O) δ 8.91 (s, 1H, H8), 7.99 (s, 1H, H8), 7.917 (s, 1H, triazole), 6.99 (t, 1H, *J*=8.42 Hz, Ph), 6.60 (t, 2H, *J*=.8.47, Ph), 5.86 (d, 1H, H-1′, *J*=3.08), 5.79 (d, 1H, H-1′, *J*=6.26), 4.66 (t, 1H, H-2′), 4.66 (t, 1H, H2′), 4.60 (t, 2H, triazole), 4.53-4.51 (m, 1H, H2′), 4.49-4.48 (m, 1H, H3′), 4.42 (t, 1H, H3′), 4.40-4.36 (m, 2H, H4′, H4′), 4.34-4.29 (m, 2H, H5′, H5′), 4.25-4.22 (m, 2H, H5″, H5″), 3.91 (s, 3H, CH_3), 3.78 (s, 6H, OCH_3), 3.80-3.75 (m, 2H, CH_2-Ph), 3.76-3.63 (m, 4H, NH-CH_2-CH_2-triazole), 2.25-.2.21 (m, 2H, CH_2-CH_2-CH_2-Ph). ^{31}P NMR (243 MHz, D_2O): -14.70 (2P, Pα,γ), -26.21 (1P, Pβ); HRMS (ES+) m/z: (M+H)+: 1092.23777, calculated for $C_{36}H_{49}N_{13}O_{21}P_3^+$: 1092.23733.

^{1}H NMR

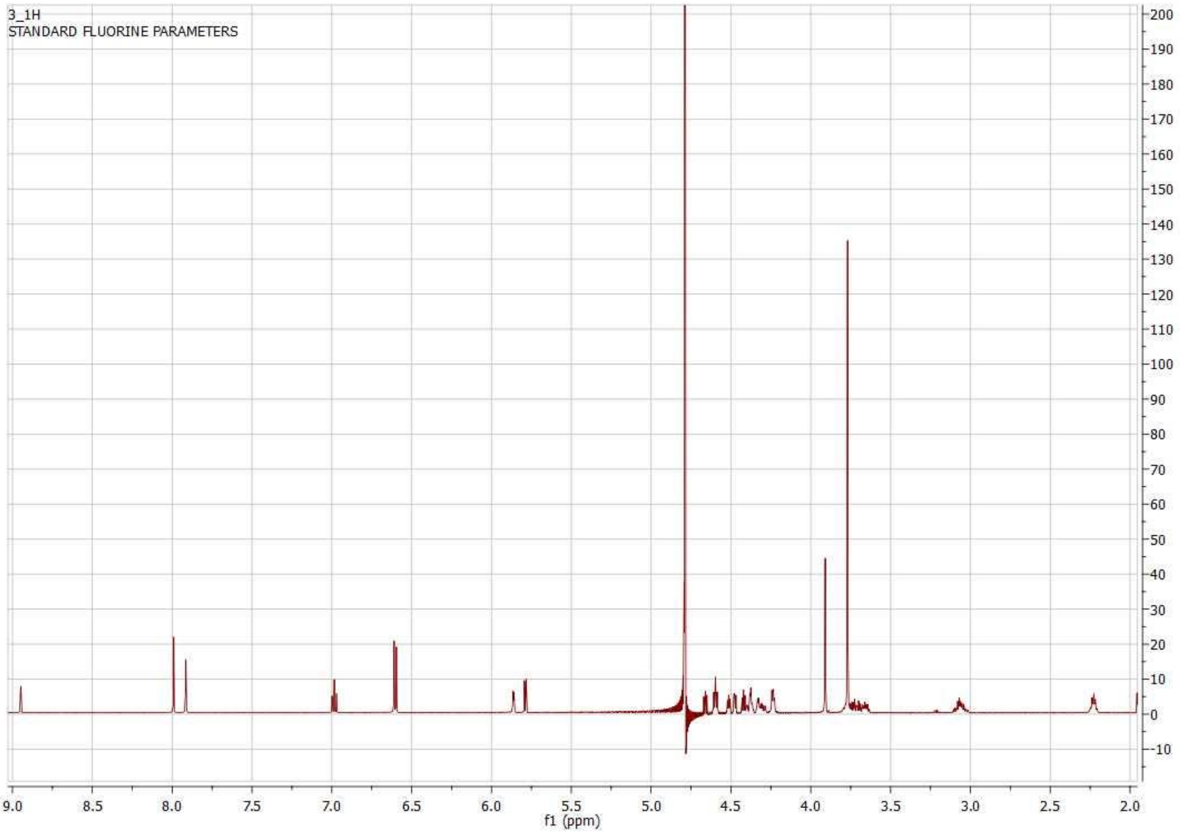

^{31}P NMR

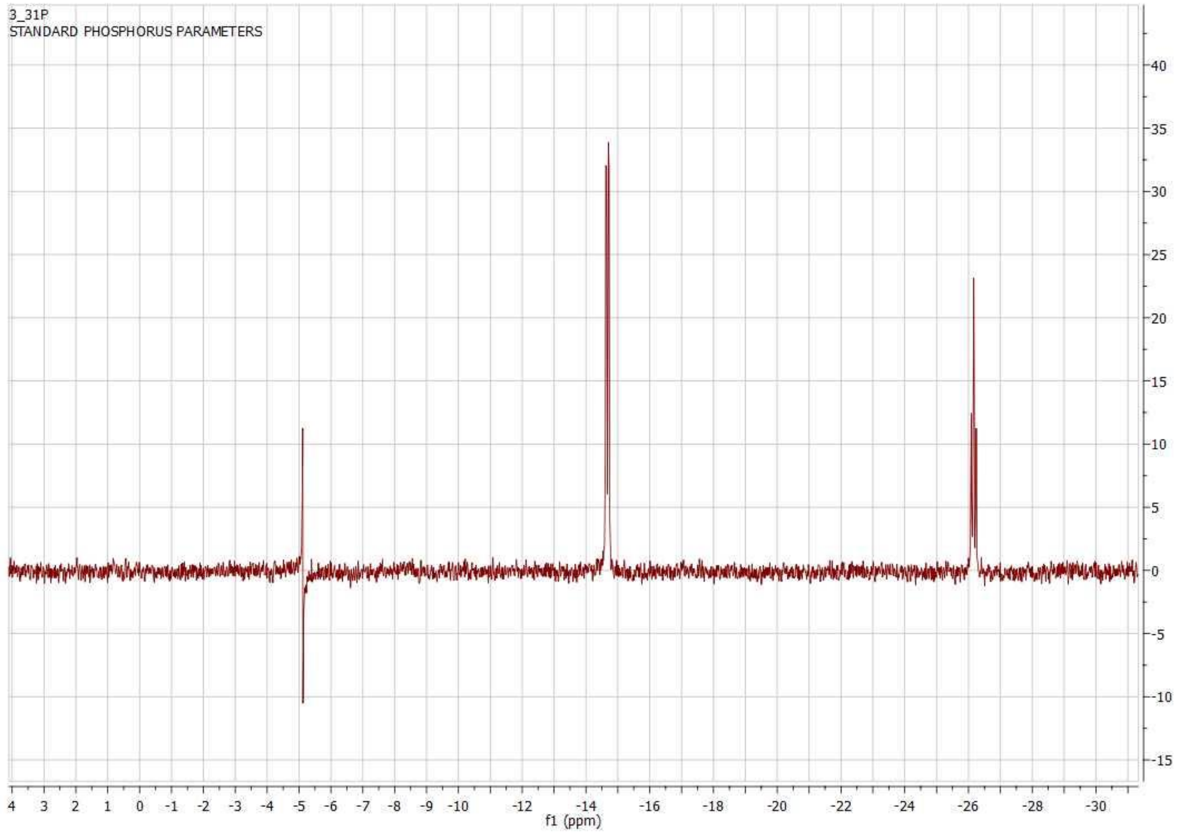

HRMS (ES+)

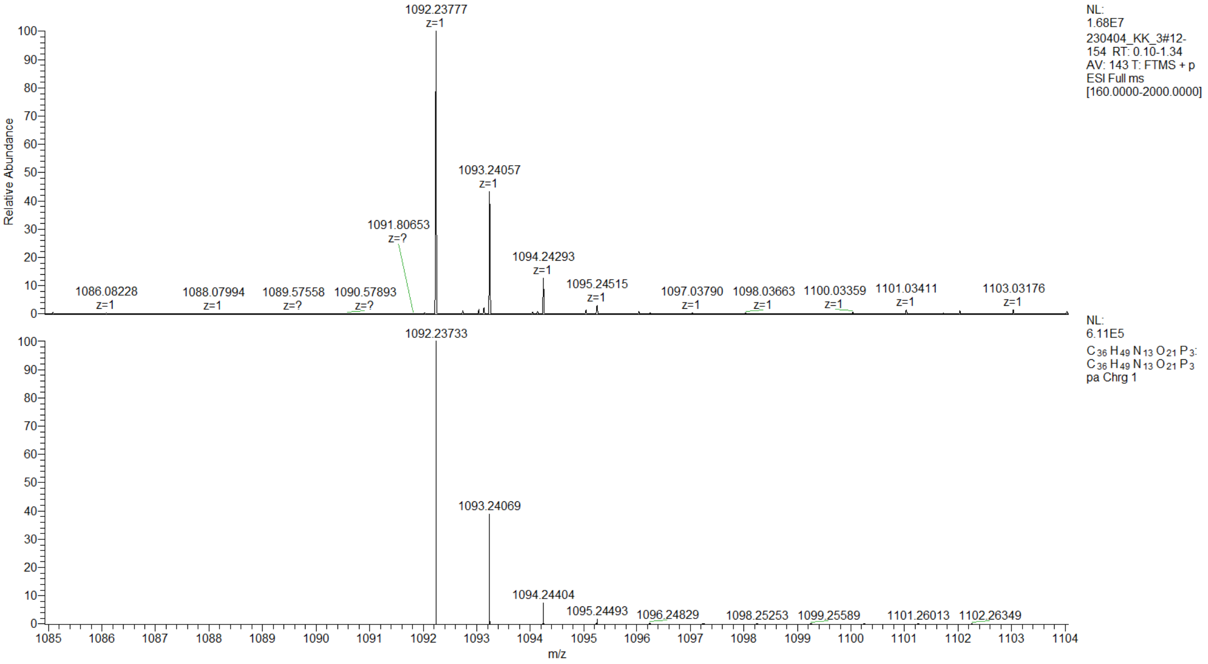

P1-N2-{1-[2-(2,6-dimethoxyphenoxy)propyl]-1H-1,2,3-triazol-4-yl}butylene-7-methylguanosine-P3-guanosine 5′,5′-triphosphate (3)

2,1 mg (0.018 mmoL), 32 %, ammonium salt; ^{1}H NMR (600 MHz, DMSO-d_6) δ 8.10 (s, 1H, H8), 7.99 (s, 1H, triazol), 6.91 (t, 1H, *J*=8.82 Hz, Ph), 6.45 (t, 2H, *J*=8.46, Ph), 5.80 (d, 1H, H-1′, *J*=2.09), 5.77 (d, 1H, H-1′, *J*=6.25), 4.65-4.57 (m, 6H, H2′, H2′, CH_2,-triazole-CH_2), 4.49-4.47 (m, 2H, H3′, H3′), 4.45-4.42 (m, 2H, H4′, H4′), 4.39-4.30 (m, 4H, H5′, H5′, H5″, H5″), 3.94 (s, 3H, CH_3), 3.87-3.75 (m, 2H, CH_2-CH_2-CH_2-Ph), 3.66 (s, 6H,OCH_3). ^{31}P NMR (243 MHz, DMSO-d_6): -15.65 (2P, Pα, γ), -26.39 (1P, Pβ); HRMS (ES+) m/z: (M+H)+: 1120.26900, calculated for $C_{38}H_{53}N_{13}O_{21}P_3^+$: 1120.26863.

^{1}H NMR

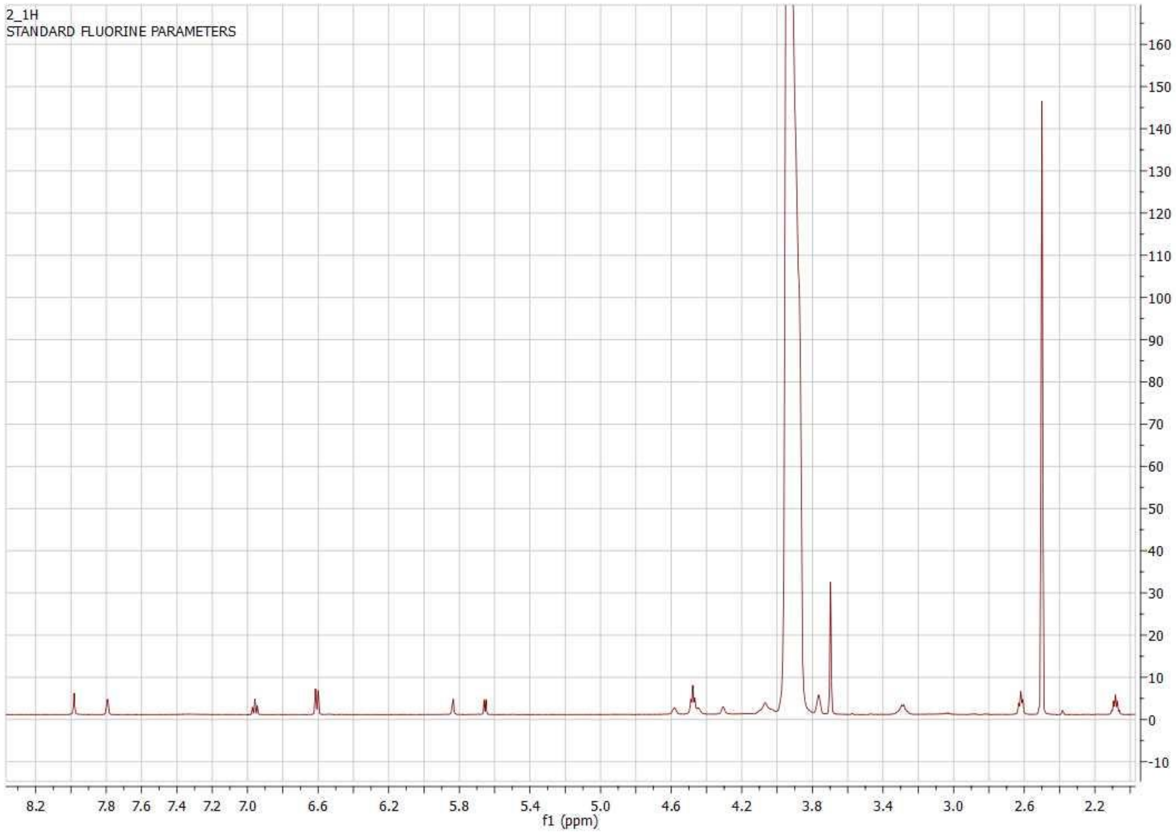

^{31}P NMR

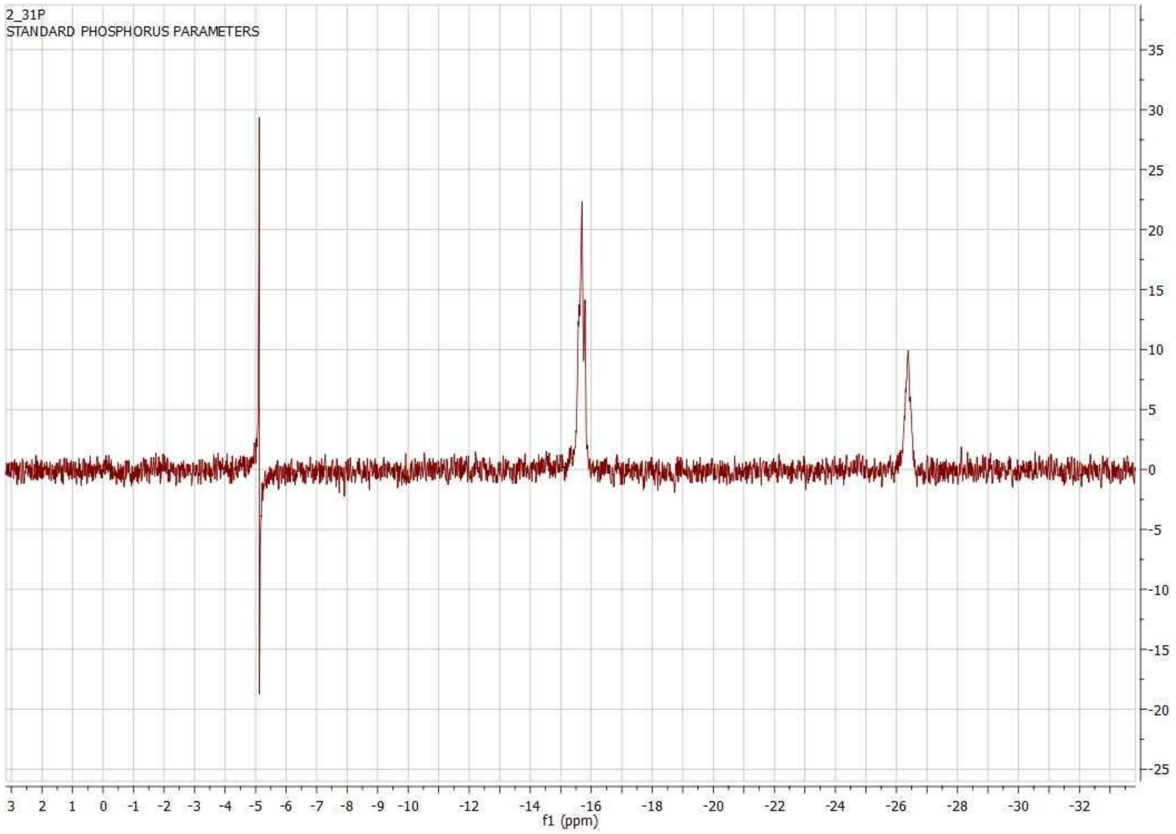

HRMS (ES+)

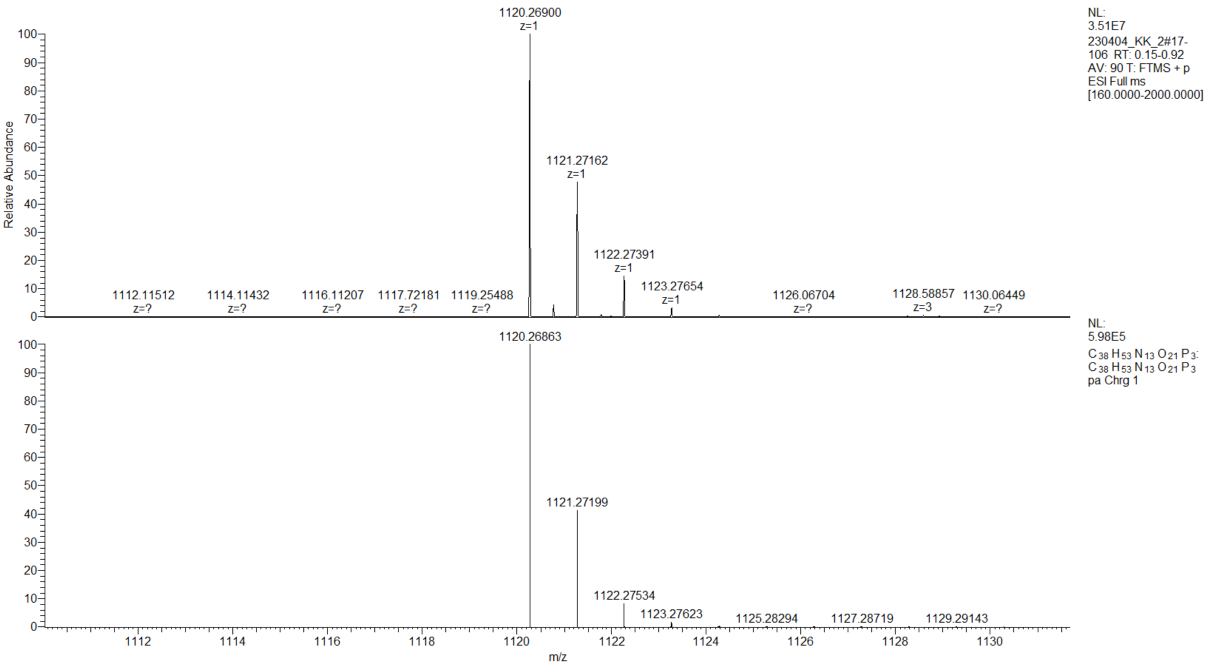

P1-N2-benzyl-7-methylguanosine-P4-guanosine 5′,5′-tetraphosphate (4)
4.9 mg (0.05 mmoL), 7%, ammonium salt; ^{1}H NMR (600 MHz, D_2O) δ 8.02 (s, 1H, H8), 7.45-7.31 (m, 5H, Ph), 5.97 (d, 1H, *J*=3.2, H1′), 5.81 (d, 1H, *J*=6.33, H1′), 4.71-4.69 (m, 1H, H2′), 4.64-4.55 (m, 3H, H2′, NH-CH_2-Ph), 4.52–4.51 (m, 1H, H3′), 4.44–4.42 (m, 1H, H3′), 4.41-4.37 (m, 2H, H4′, H4′), 4.40-4.24 (m, 4H, 5′, 5′, 5″, 5″), 4.06 (s, 3H, CH_3). ^{31}P NMR (243 MHz, D_2O): -14.49 (2P, Pα,δ), -26.14 (2P,Pβ,γ); HRMS (ES+) m/z: (M+H)+: 973.10841, calculated for $C_{28}H_{37}N_{10}O_{21}P_4^{+}$: 973.10797

^{1}H NMR

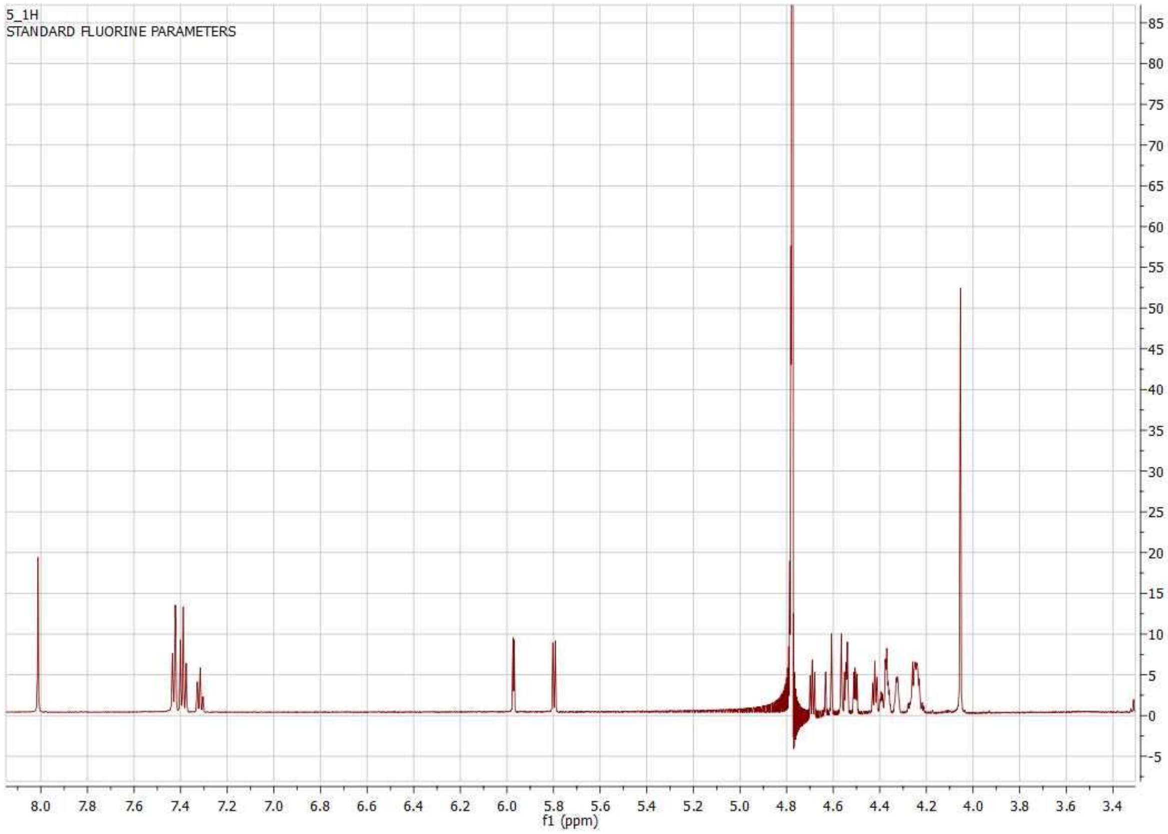

^{31}P NMR

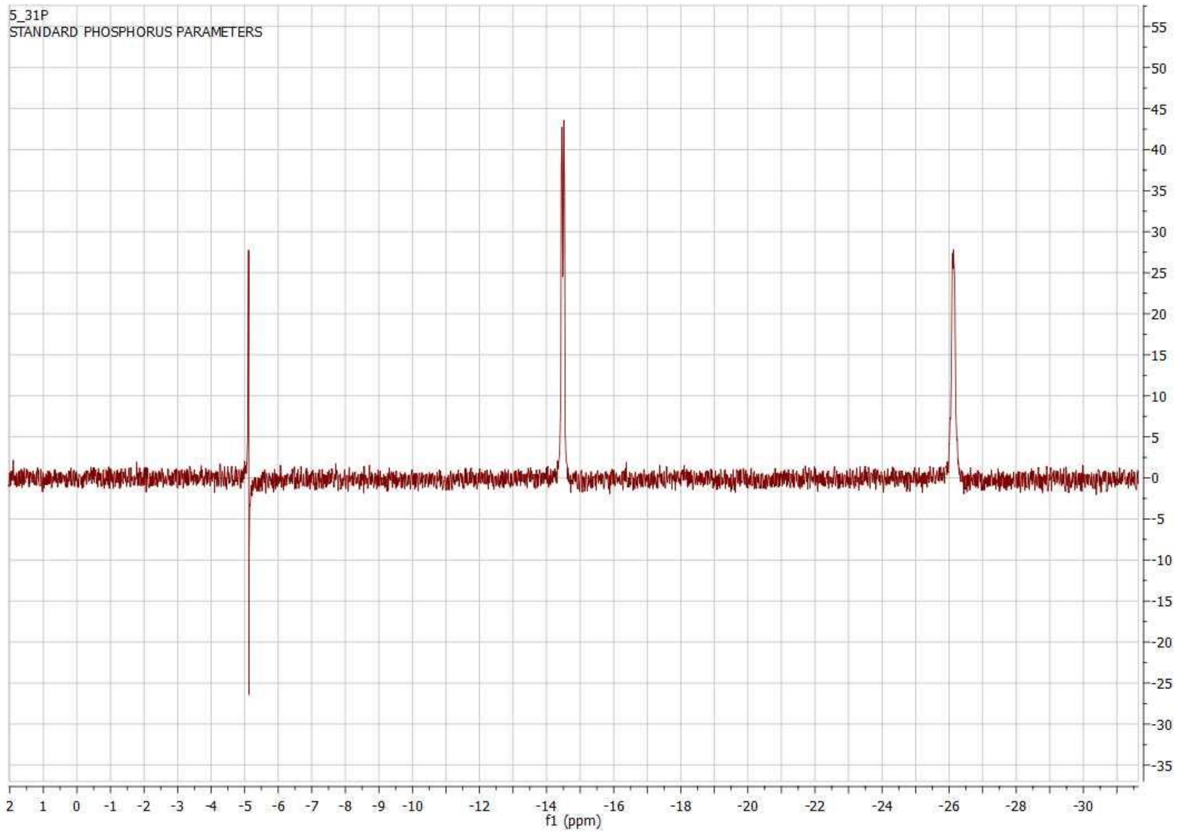

HRMS (ES+)

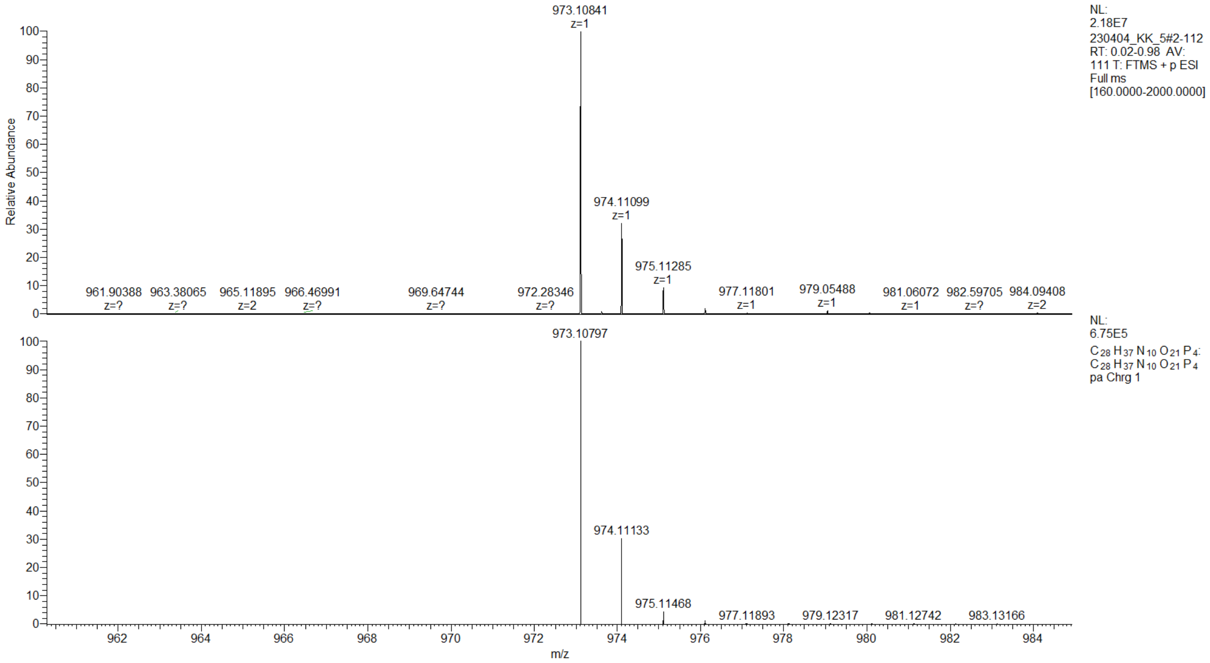

P1-N2-(p-chlorobenzyl)-7-methylguanosine-P4-guanosine 5′,5′-tetraphosphate (5)
4.9 mg (0,0095 mmoL), 20 %, ammonium salt; ^{1}H NMR (600 MHz, D_2O) δ 8.02 (s, 1H, H8), 7.38-7.35 (m, 4H, Ph), 5.96 (d, 1H, *J*=8.3, H1′), 5.79 (d, 1H, *J*=2.45, H1′), 4.70-4.68 (m, 1H, H2′), 4.59-4.50 (m, 4H, H-2′, H3′, NH-CH_2-Ph), 4.38 (m, 3H, H4′, H4′, H3′), 4.33-4.24 (m, 4H, H5′, H5′, H5″, H5″), 4.06 (s, 3H, CH_3). ^{31}P NMR (243 MHz, D_2O): -14.48 (2P, Pα,δ), -26.23 (2P, Pβ,γ); HRMS (ES+) m/z: (M+H)+: 1007.07017, calculated for $C_{28}H_{36}ClN_{10}O_{21}P_4^{+}$: 1007.06900.

^{1}H NMR

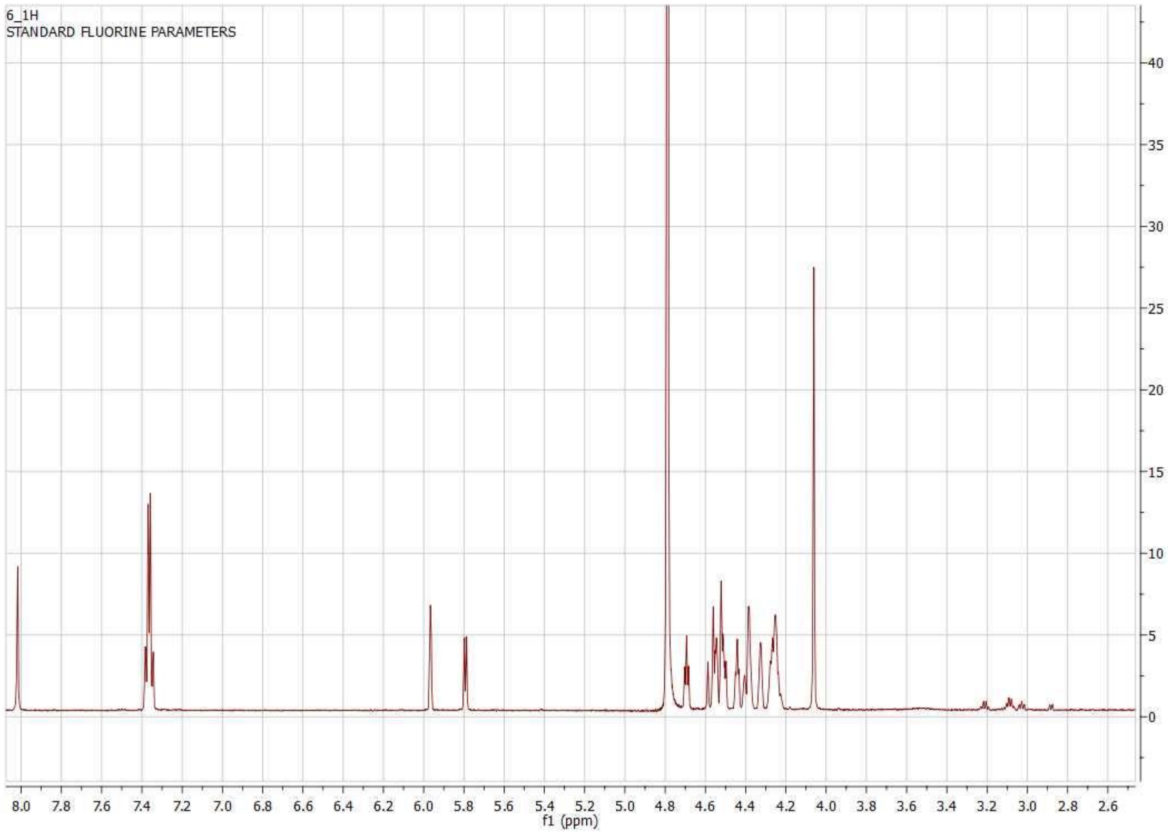

^{31}P NMR

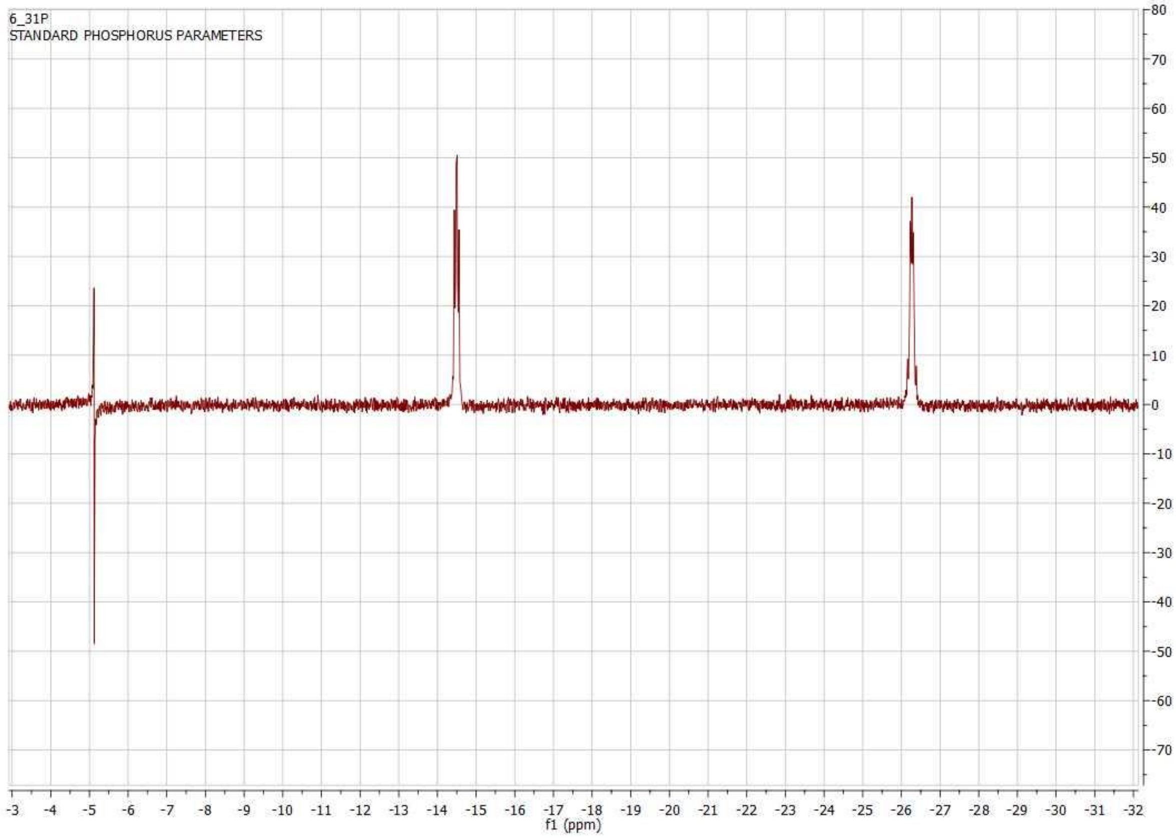

HRMS (ES+)

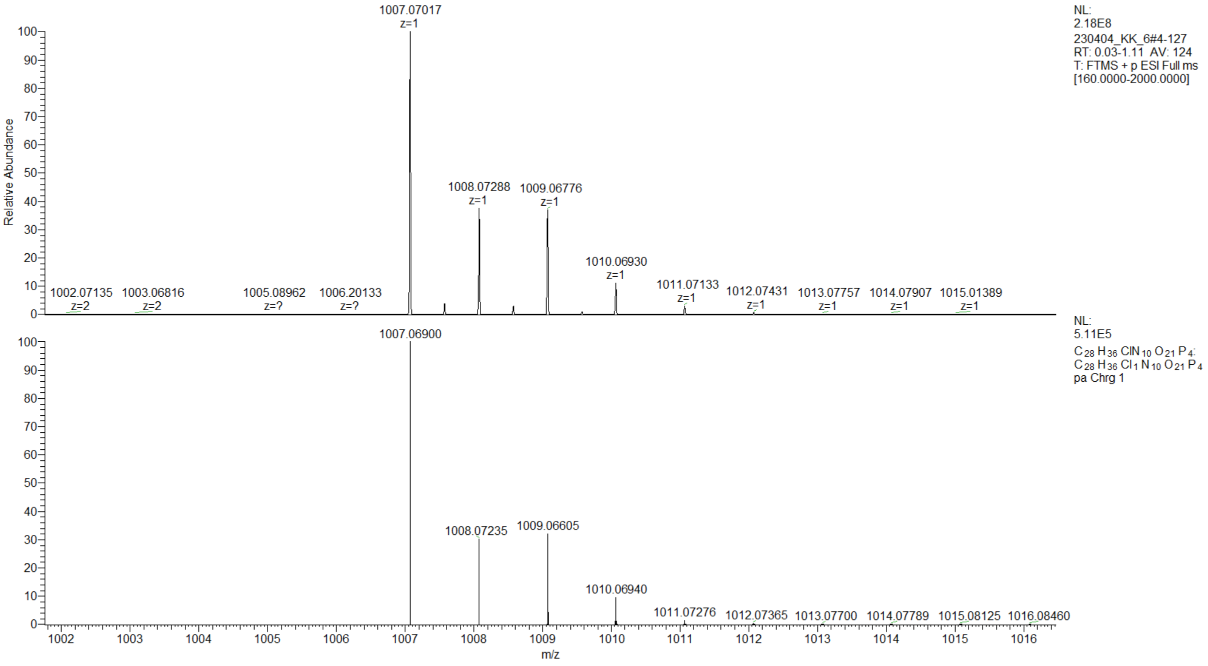

Synthetic procedure for dinucleotide cap analogue modified at the N2 position and β phosphate

Synthesis of N2-benzyl-7-methylguanosine-5′-O-(2-thiophosphate) ($bn^2m^7GDP_S$)

To dissolved of imidazole derivative of 7-methylguanosine 5′-monophosphate modified at the N2 position (obtained according to (Piecyk et al. 2020)) (1 eq.) in anhydrous DMF, anhydrous $ZnCl_2$ (6 eq.) and thiophosphate triethylamonium salt (5 eq.) were added and allowed to stir vigorously for 20 min at RT. The reaction was quenched by adding an aqueous solution of EDTA in disodium salt (73 mg/1 mL). The resulting thiodiphosphate semi-product was isolated from the mixture on DEAD-Sephadex (gradient elution 0–1.0 M TEAB). Yield: 17 mg as TEA salt (41 %, 0,0025mmol).

Synthesis of P1-N2-benzyl-7-methylguanosine-P2-thiophosphate-P3-guanosine 5′,5′-triphosphate (6)

To dissolved of imidazole derivative of guanosine 5′-monophosphate (2 eq.) in anhydrous DMF, anhydrous $ZnCl_2$ (6 eq.) and $bn^2m^7GDP_S$ as TEA salt (1 eq.)were added and allowed to stir vigorously for 24h at RT. The reaction was quenched by adding an aqueous solution of EDTA in disodium salt (73 mg/1 mL). The resulting dinucleotide product (**6**) was isolated from the mixture on DEAD-Sephadex (gradient elution 0–1.0 M TEAB) and purified by semi-preparative RP HPLC (gradient elution 0–50% MeOH in 0.05 M ammonium acetate buffer pH 5.9) to afford—after evaporation and repeated freeze-drying from water—ammonium salt of dinucleotide cap analogues. The reaction yields 23 mg as TEA salt (19%, 0,022 mmol)

^{1}H NMR (400 MHz, D_2O) δ: 8.01; 7.99 (s, $2X_1H$, H8), 7.45-7.31 (m, $2X_5H$, Ph), 5.94-5.92 (2xd, $2X_1H$, H1′), 5.80-5.79 (2xd, $2X_1H$, H1′), 4.70-4.25 (m, 20H with H2′, H3′, H4′, H5′, H5″ and 4H with CH_2-Ph), 4.06; 4.06 (2xs, $2X_3H$, CH_3) ^{31}P NMR (162 MHz, D_2O) δ -15.4 to 1-15.56 ($2X_2P$, Pα, γ), +26.91; 26.83 ($2X_1P$, Pβ-SH); HRMS: (ES+) m/z: (M+H)+: 909.11922, calculated for $C_{28}H_{36}N_{10}O_{17}S_1P_3^+$: 909.11880

^{1}H NMR

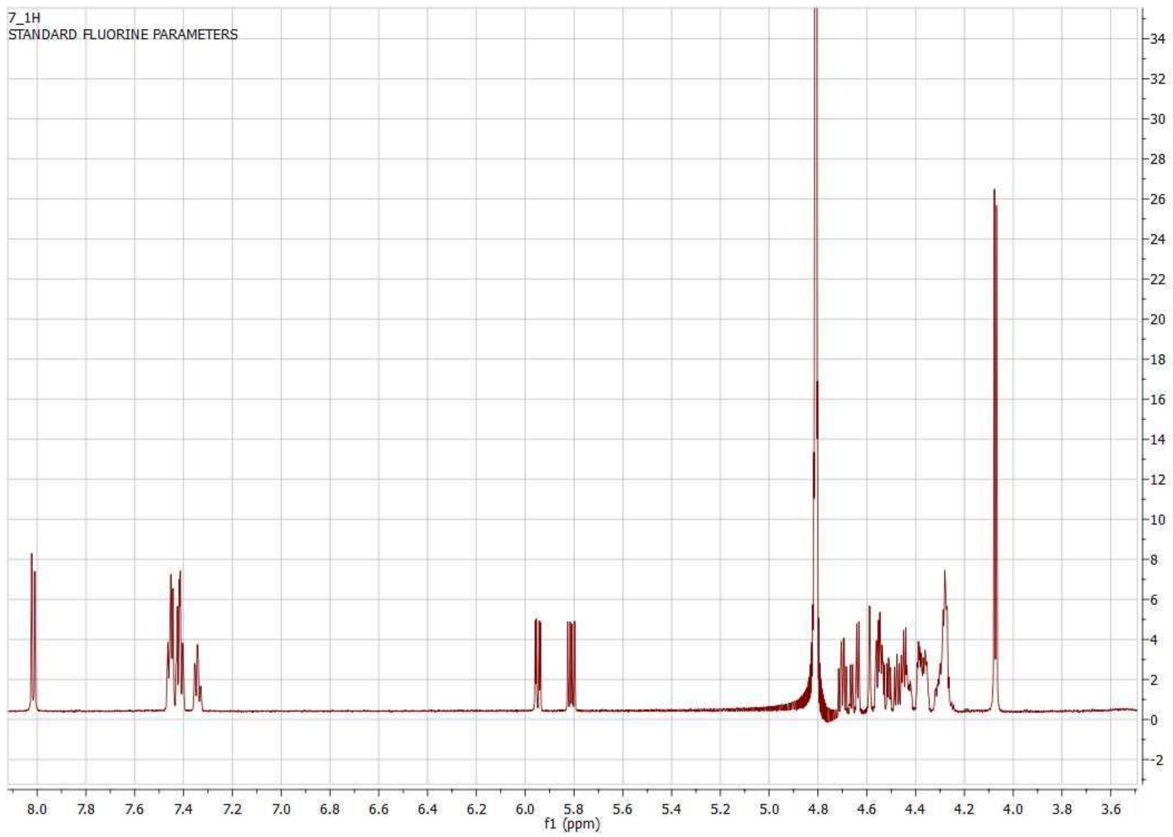

^{31}P NMR

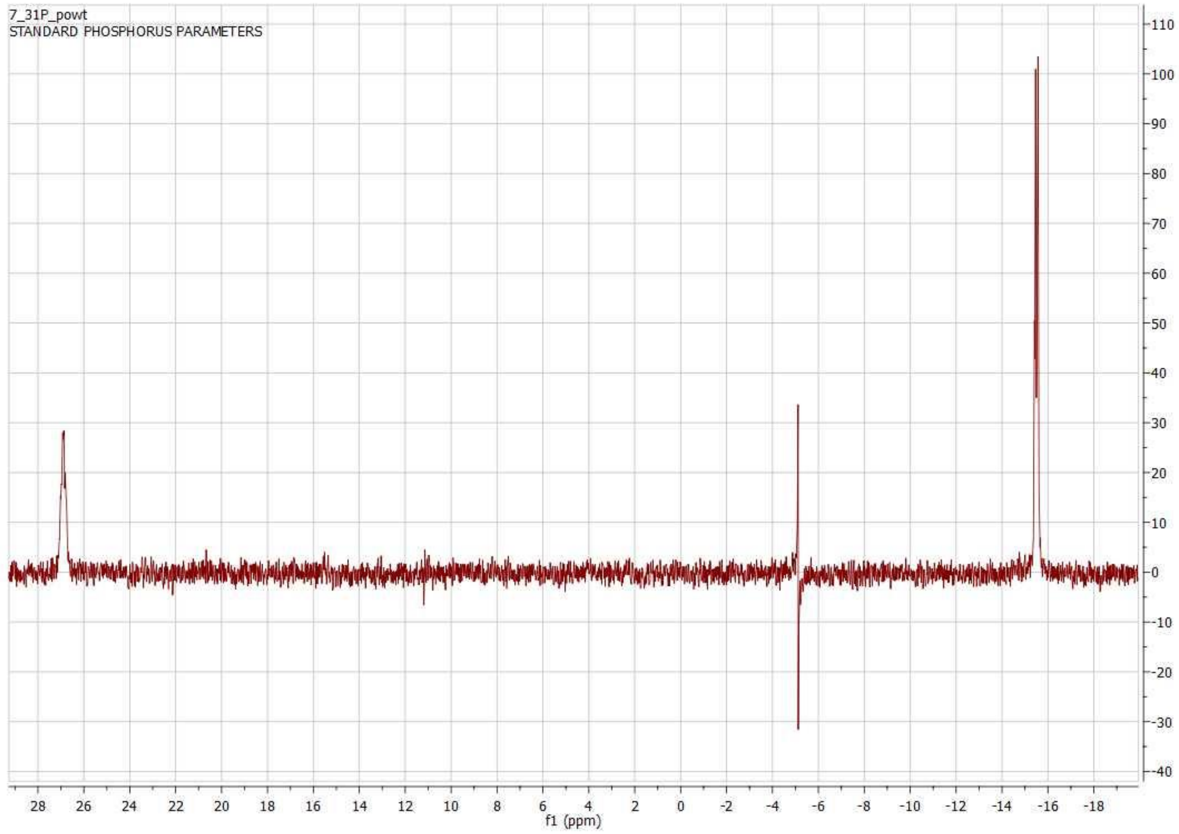

HRMS (ES+)

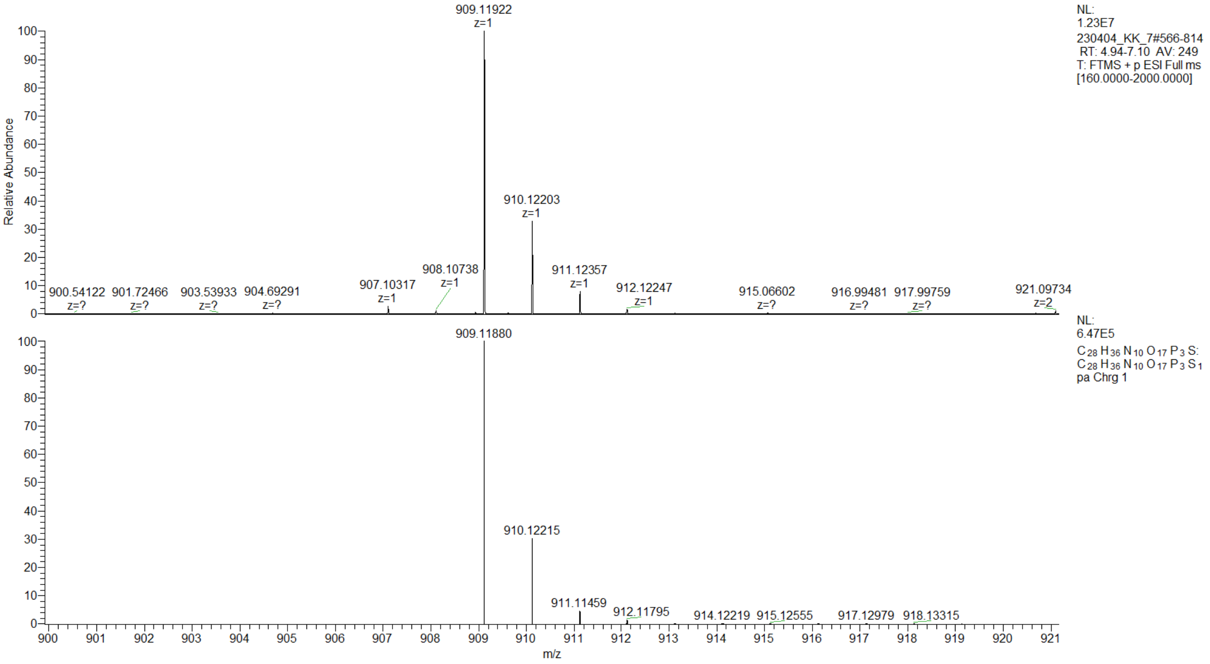

General procedure used to obtain trinucleotide cap analogues containing A_m

The dinucleotide (pA_mpG) synthesis was performed in a solution, using amidophosphite method. The conjugation reaction was performed using (1 eq.) of 5′-O-DMT-2′-O-methyl-3′-O-phosphoramide and (1 eq.) of N2-isobutyryl-2′,3′-isopropylidene-guanosine in the presence of 0.40 M 5-(benzylthio)-1-H-tetrazole in acetonitrile. The reaction was performed over 4 hours at room temperature, under argon atmosphere. After 4h, the mixture was cooled to 4ºC and 0.1M iodine in pyridine was added and mixed for 1 hour at room temperature. The reaction mixture was extracted with dichloromethane and washed with brine. The obtained organic layer was dried and evaporated. Crude compound was dissolved in DCM containing dichloroacetic acid (5% solution) and mixed until fully detritylation. Reaction was quenched by extracted with dichloromethane and 10% $NaHCO_3$ in water. Organic layer was dried and evaporated and purified using flash chromatography on silica gel, using gradient elution (0→5% methanol in dichloromethane). The purified compound was dissolved in 20% aqueous TFA solution and mixed at RT for 4 hours. The mixture was evaporated under vacuum and evaporated 6 times with methanol. The raw nucleotide was crystallised from diethyl ether. The precipitate was filtered, washed with diethyl ether and dried in a vacuum dessicator over phosphorus pentaoxide. In the last stage, the dinucleotide was phosphorylated at the 5′-OH position using the standard Yoshikawa method (Yoshikawa, Kato, and Takenishi 1969). POCl3 (7 eq.) was added to dissolved and cooled dinucleotide (1 eq.) in TMP (170 eq.) and allowed to stir vigorously for 6h at -19. The obtained product had its protection removed with ammonia, was evaporated and purified using ion exchange chromatography on DEAE-Sephadex (A-25, HCO3- form) using a linear gradient of triethylammonium bicarbonate (TEAB), pH 7.5 in water. The fractions containing the desired product have been combined, evaporated and lyophilised to obtain the TEA salt of the product as white powder.

The triethylammonium salt of pA_mpG (1 eq.), imidazole derivative of N2-modified diphosphate (2 eq.) and anhydrous $ZnCl_2$ (25 eq.) were dissolved in anhydrous DMSO. The mixture was mixed at RT for 24 hours and the reaction was then stopped by adding aqueous EDTA solution(25mg/ml). The product was isolated using ion exchange chromatography on DEAE Sephadex (gradient elution using 0-1.2 M TEAB) and purified by semi-preparative RP-HPLC (gradient elution 0–50% MeOH in 0.05 M ammonium acetate buffer pH 5.9) to afford—after evaporation and repeated freeze-drying from water—ammonium salt of trinucleotide cap analogues. The reaction yield varied between 10 and 40%.

bn^{2}m^{7}GpppA$_{m}$pG (7)

HRMS: (ES+) m/z: (M+H)+: 1236.21098 calculated for $C_{39}H_{50}N_{15}O_{24}P_{4}^{+}$: 1236.20981

HRMS (ES+)

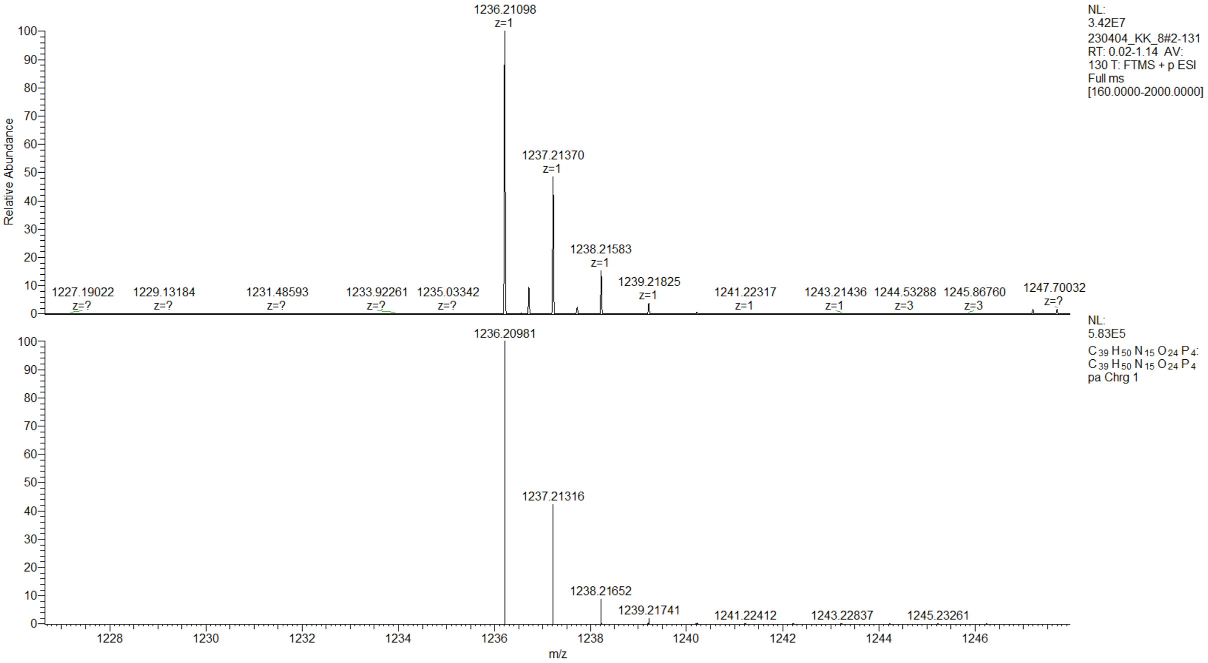

(4-Cl-bn)^{2}m^{7}GpppA$_{m}$pG (8)

HRMS: (ES+) m/z: (M+H)+: 1270.17113 calculated for $C^{39}H^{49}N^{15}O^{24}Cl_{1}P_{4}^{+}$: 1270.17084

HRMS (ES+)

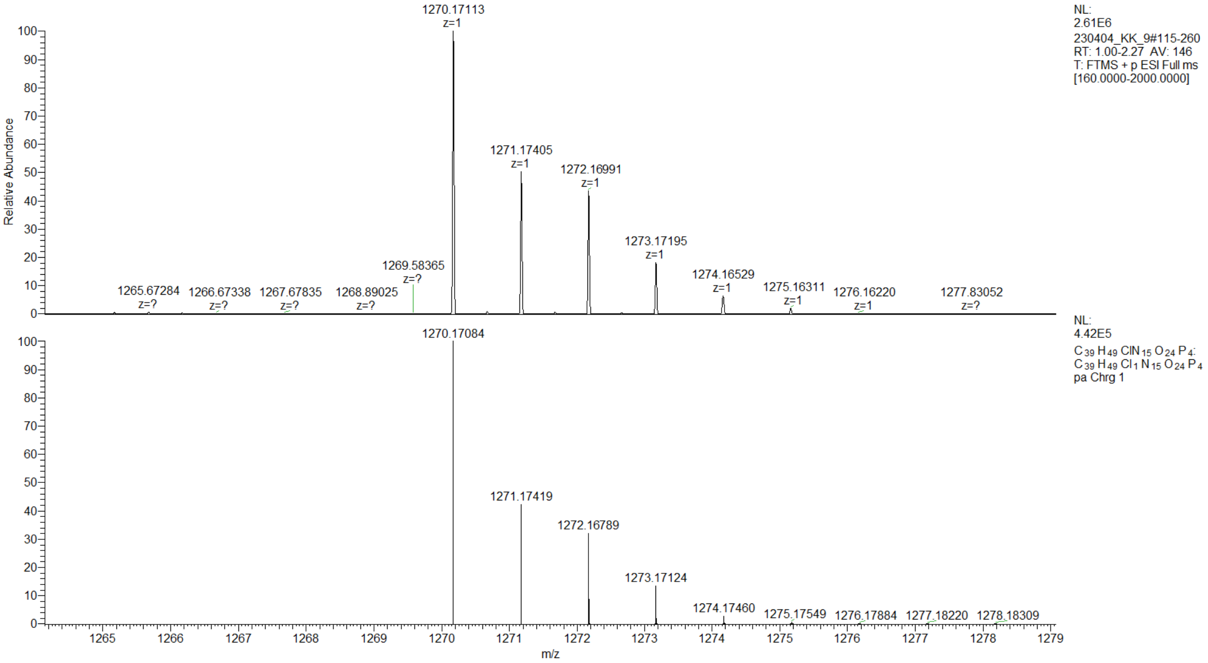

(4-bn-isx)^{2}m^{7}GpppA$_m$pG (9)

HRMS: (ES+) m/z: (M+H)+: 1303.21658 calculated for $C_{42}H_{51}N_{16}O_{25}P_4^+$: 1303.21562

HRMS (ES+)

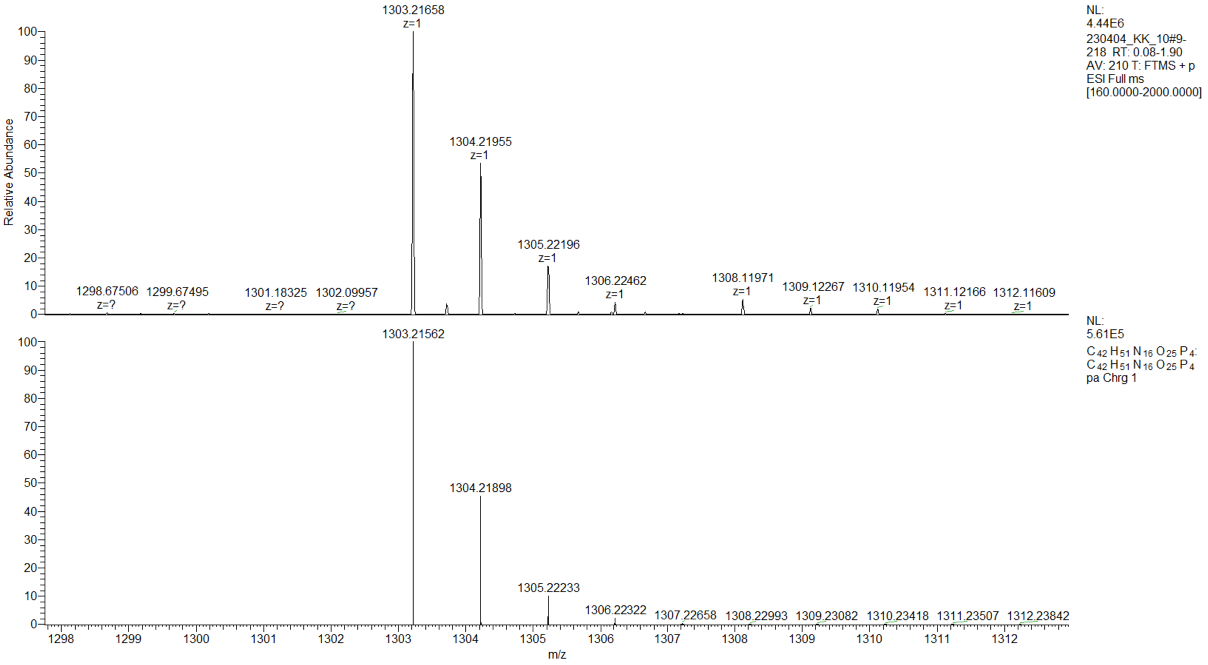

Supplementary Figure 1

A

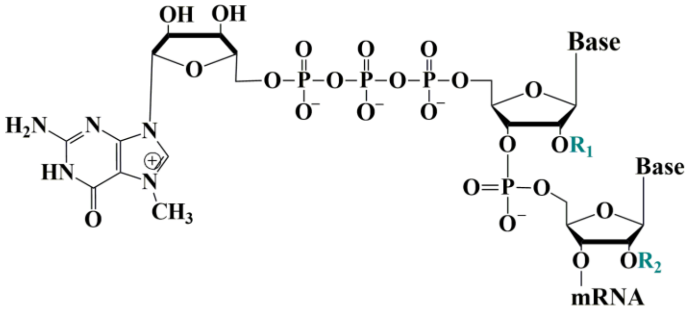

cap 0: R_1 = -H, R_2 = -H

cap 1: R_1 = $-CH_3$, R_2 = -H

cap 2: R_1 = $-CH_3$, R_2 = $-CH_3$

B

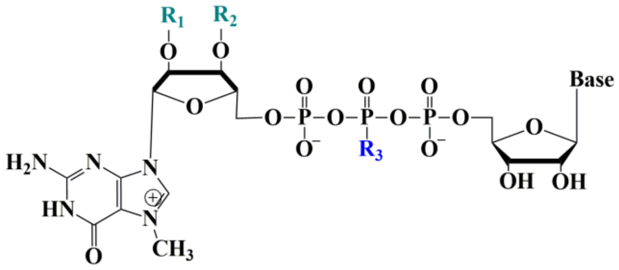

ARCA 2': R_1 = $-CH_3$, R_2 = -H, R_3 = $-O^-$

ARCA 3': R_1 = -H, R_2 = $-CH_3$, R_3 = $-O^-$

B-S-ARCA: R_1 = $-CH_3$, R_2 = -H, R_3 = $-S^-$

Gels depicting mRNA capping and decapping with hNudt16

Gel fragments where 24-26-nt RNAs after DNAzyme trimming are visualised are framed in red and these bands were used for densitometric analysis. ✦ indicates 34 – 36-nt RNAs that did not undergo DNAzyme trimming, ★ shows bands that correspond to undigested DNAzyme. All other bands are due to the premature termination of transcription by T7 polymerase.

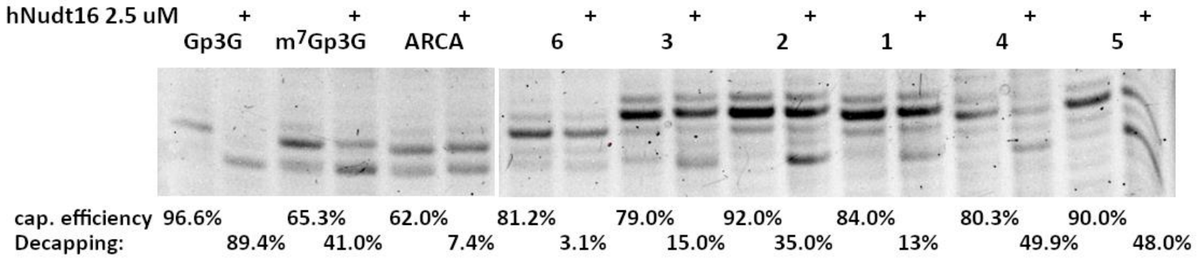

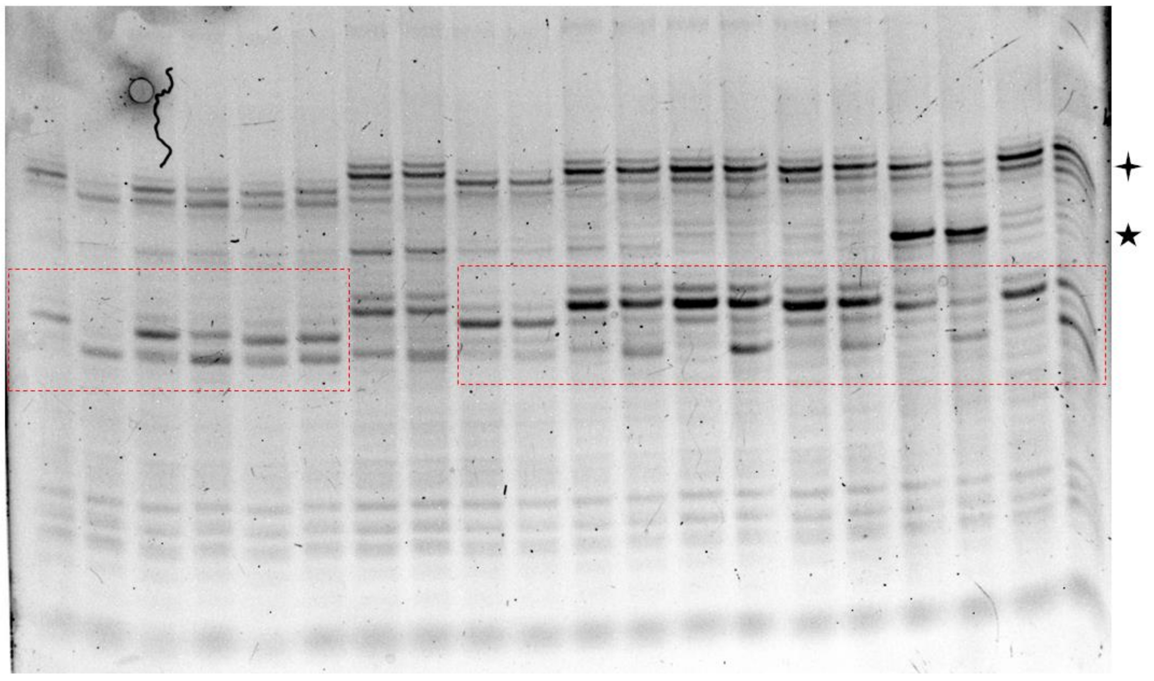

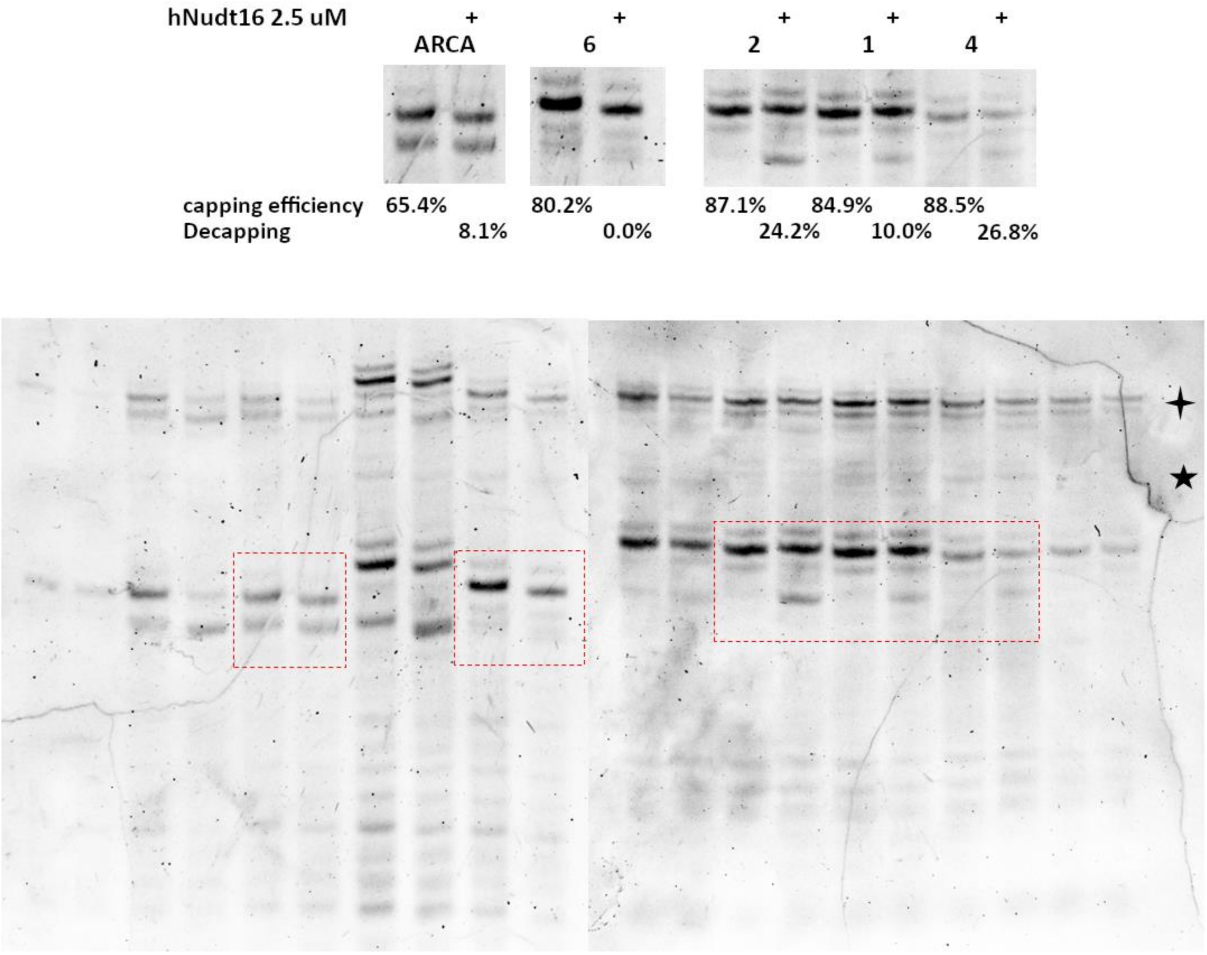

hNudt16 2.5 uM
+
+
+
+
+
ARCA
6
2
1
4
capping efficiency
65.4%
80.2%
87.1%
84.9%
88.5%
Decapping
8.1%
0.0%
24.2%
10.0%
26.8%

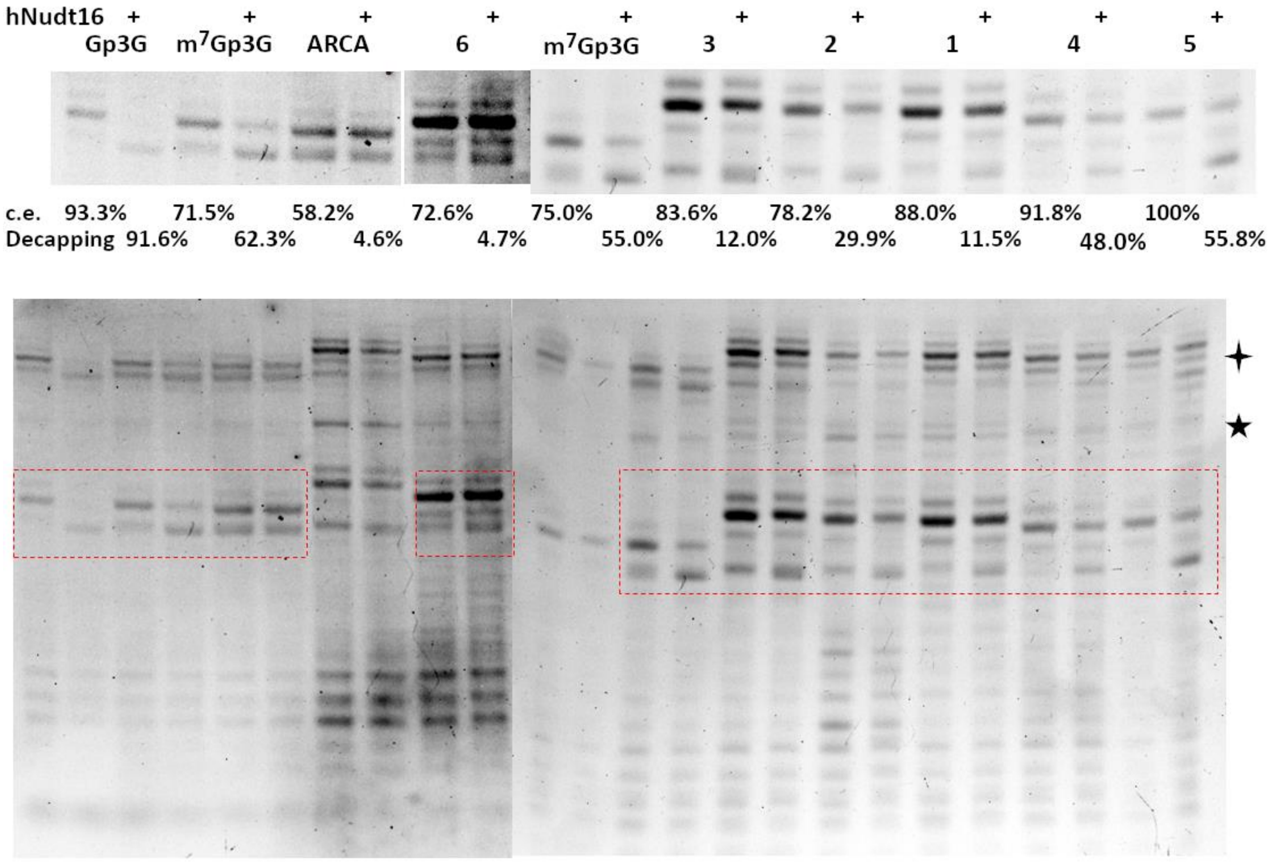
hNudt16 + + + + + + + + + +
Gp3G m7Gp3G ARCA 6 m7Gp3G 3 2 1 4 5
c.e. 93.3% 71.5% 58.2% 72.6% 75.0% 83.6% 78.2% 88.0% 91.8% 100%
Decapping 91.6% 62.3% 4.6% 4.7% 55.0% 12.0% 29.9% 11.5% 48.0% 55.8%

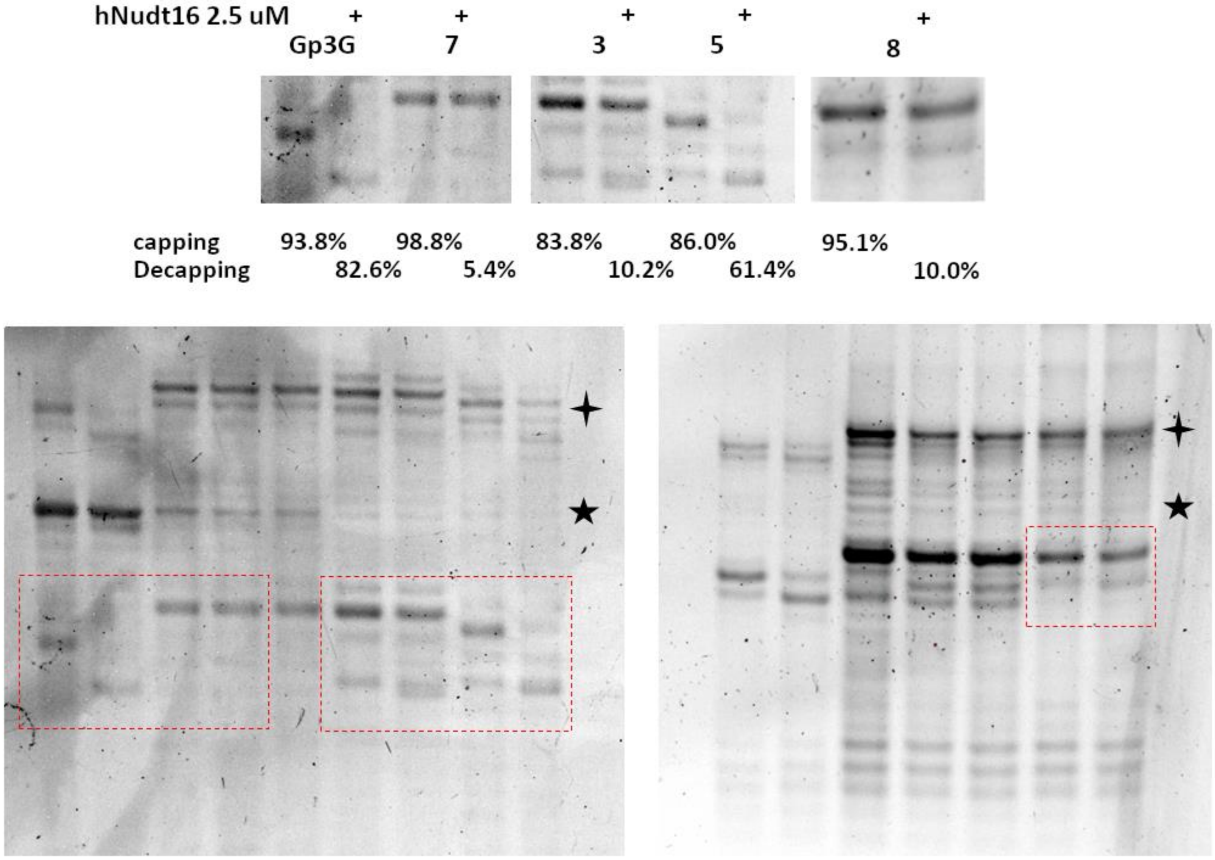
hNudt16 2.5 uM + + + + +
Gp3G 7 3 5 8
capping 93.8% 98.8% 83.8% 86.0% 95.1%
Decapping 82.6% 5.4% 10.2% 61.4% 10.0%

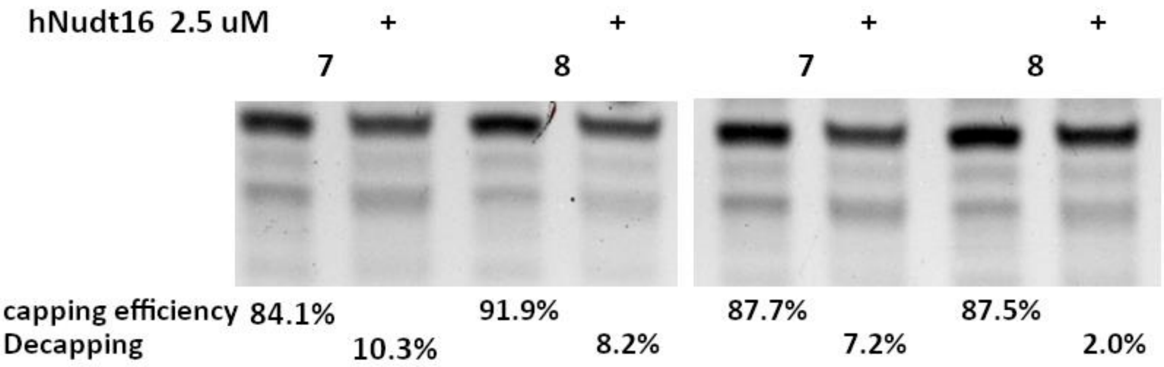

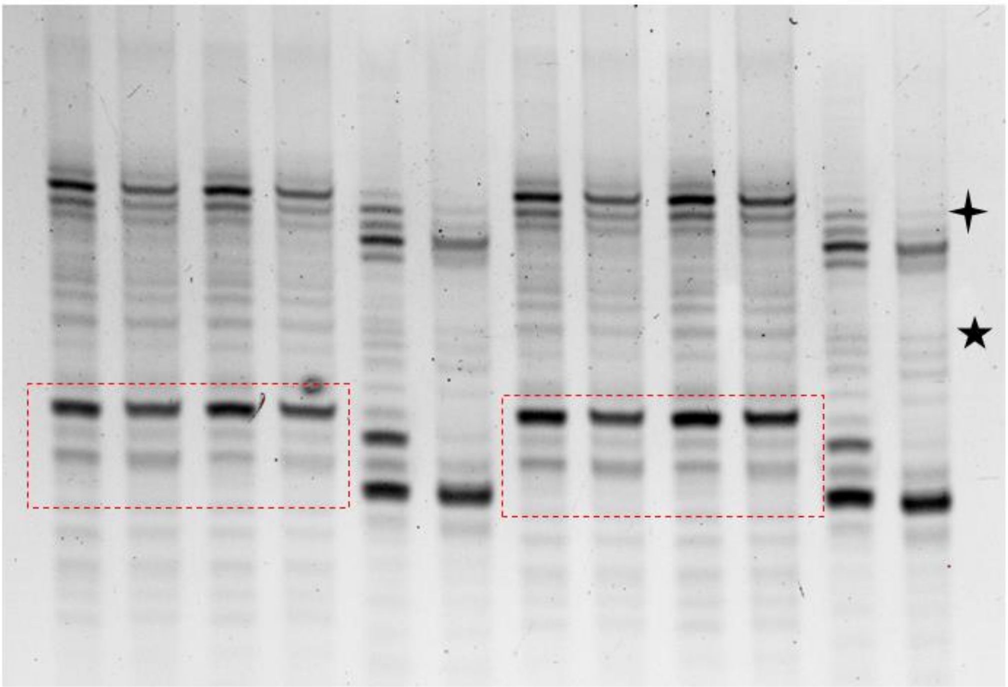

References

Eisenführ, Alexander, Paramjit S. Arora, Gerhard Sengle, Leo R. Takaoka, James S. Nowick, and Michael Famulok. 2003. “A Ribozyme with Michaelase Activity: Synthesis of the Substrate Precursors.” *Bioorganic & Medicinal Chemistry* 11(2):235–49.

Grzela, Renata, Karolina Piecyk, Anna Stankiewicz-Drogon, Paulina Pietrow, Maciej Lukaszewicz, Karol Kurpiejewski, Edward Darzynkiewicz, and Marzena Jankowska-Anyszka. 2022. “N2 Modified Dinucleotide Cap Analogues as a Potent Tool for MRNA Engineering.” *RNA* 29(2):rna.079460.122.

Kocmik, Ilona, Karolina Piecyk, Magdalena Rudzinska, Anna Niedzwiecka, Edward Darzynkiewicz, Renata Grzela, and Marzena Jankowska-Anyszka. 2018. “Modified ARCA Analogs Providing Enhanced Translational Properties of Capped MRNAs.” *Cell Cycle* 15384101.2018.1486164.

Piecyk, Karolina, Paulina Pietrow, Thomas Arnold, Remigiusz Worch, Nadejda L. Korneeva, and Marzena Jankowska-Anyszka. 2020. “Effect of HIV-1 TAT Peptide Fusion on 5′ MRNA Cap Analogs Cell Membrane Permeability and Translation Inhibition.” *Bioconjugate Chemistry* 31(4):1156–66.

Yoshikawa, Masaharu, Tetsuya Kato, and Tadao Takenishi. 1969. “Studies of Phosphorylation. III. Selective Phosphorylation of Unprotected Nucleosides.” *Bulletin of the Chemical Society of Japan* 42(12):3505–8.
